# Inclusion of Jack Mackerel Meal in Low‐Fish Meal Diets: Impacts on the Growth and Feed Utilization of Red Sea Bream (*Pagrus major*) and Economic Analysis

**DOI:** 10.1155/anu/5589868

**Published:** 2026-07-02

**Authors:** Yu Jin Sim, Sung Hwoan Cho

**Affiliations:** ^1^ Department of Convergence Interdisciplinary Education of Maritime and Ocean Logistics, Korea Maritime and Ocean University, Busan, 49112, Republic of Korea, kmou.ac.kr; ^2^ Division of Convergence on Marine Science, Korea Maritime and Ocean University, Busan, 49112, Republic of Korea, kmou.ac.kr

**Keywords:** combination of animal and plant protein sources, economic profit, feed stimulant, fish meal replacer, jack mackerel meal

## Abstract

Combined animal and plant sources can replace higher levels of fish meal (FM) in fish feeds than a single source, as they compensate for the nutritional imbalances and deficiencies inherent to individual protein sources. Nevertheless, low‐FM diets often exhibit deteriorated palatability and reduced feed intake, ultimately leading to retarded growth. This experiment, therefore, aims to assess inclusion impacts of jack mackerel meal (JMM) in low FM diets substituting various levels of FM with combined meat meal (MM) and corn gluten meal (CGM) (MC) on the growth and feed utilization of red sea bream (*Pagrus major*) and economic analysis. A two‐way (FM substitution level [SL] [25% and 50%] × JMM inclusion [without or with]) ANOVA experimental design was adopted. A total of 375 juvenile (initial mean weight of 2.0 g) fish were randomly distributed into 15 flow‐through tanks. The control (Con) diet included 60% FM. In the Con diet, 25% and 50% of FM were substituted with MC, and then 24% JMM was included at the cost of FM, named the MC25, MC50, MC25J, and MC50J diets, respectively. All experimental feeds were formulated to be isoproteic (52.0%) and isolipidic (15.0%). The experimental diets were offered to fish twice daily until apparent satiation over an 8‐week period. The diets with 25% of FM substitution achieved higher weight gain (WG), specific growth rate (SGR), and feed consumption (FC) in fish than those with 50% of FM substitution. Furthermore, WG, SGR, and FC of fish fed the MC‐substituted diets with JMM inclusion were superior to those without JMM inclusion. The highest WG, SGR, and FC were attained in fish fed the MC25J diet. The MC25J diet showed the highest economic profit index (EPI), although no significant difference was detected among dietary treatments. In conclusion, the MC25J diet appears to represent the best feeding strategy to enhance the growth performance and FC in red sea bream and maximize EPI to farmers.

## 1. Introduction

Both aquaculture and capture fisheries are used to supply seafood, but only aquaculture is increasing its contribution to the seafood supply [1], as demand for seafood is growing, but resources are finite. The future of aquaculture depends on the sustainable production of aquafeeds [2]. Fish meal (FM) is commonly used as a high‐quality protein in aquafeeds because of its balanced amino acid (AA) profiles, high palatability, and outstanding digestibility [3, 4]. Nevertheless, the limited availability and rising cost of FM, driven by the expansion of global aquaculture, limit the sustainable development of aquaculture [3, 4]. Hence, feed nutritionists are actively seeking reliable, cost‐effective, and high‐quality protein sources to substitute for FM.

Meat meal (MM) is produced by removing lipids and then heating and pressurizing the nonedible parts of mammalian livestock and poultry during the slaughtering process [5]. It is classified as a grade‐feed ingredient with less than 75% crude protein (CP) [6] and pet‐grade MM with more than 80% CP [7]. Specifically, pet‐grade MM has a low price (USD 1.05/kg MM and USD 1 = 1330 KRW) and comparable or higher digestibility than FM (USD 2.41/kg FM) in feeds for juvenile silver perch (*Bidyanus bidyanus*) [8] and juvenile and grower rockfish (*Sebastes schlegeli*) [9]. Several studies have demonstrated that pet‐grade MM can replace FM in diets up to 40% for juvenile red sea bream (*Pagrus major*) [7] and olive flounder (*Paralichthys olivaceus*) [10]. Therefore, pet‐grade MM holds significant potential as a sustainable alternative to FM due to its cost‐effectiveness and high digestibility.

Corn gluten meal (CGM), an agricultural by‐product of the corn starch manufacturing industry [11], contains over 60% CP with low levels of antinutritional factors and fiber [12]. Therefore, CGM has been utilized as an FM replacer in the feeds of a variety of carnivorous fish, such as red sea bream [13], spotted rose snapper (*Lutjanus guttatus*) [12], and turbot (*Scophthalmus maximus*) [14]. In particular, CGM can replace up to 20% of FM in a 60% FM‐basal feed without adversely affecting the growth of red sea bream [13].

While individual animal and plant protein sources have the potential to replace FM in fish diets, a single source used as an FM replacer remains limited in effectiveness due to its lower indispensable AA (IAA) content compared to FM [15, 16]. For instance, pet‐grade MM contained a lower content of IAA, except for arginine (Arg) [7, 10], and CGM contained lower histidine (His), lysine (Lys), and methionine (Met) content among IAA compared to FM [12, 17]. Preventing IAA deficiency is one of the most critical challenges in the successful use of alternatives to FM in fish feeds. However, the effectiveness of the synthetic AA in fish feeds is still controversial [18–20]. Therefore, instead of relying on the synthetic AA in diets, using combined animal and plant proteins could enhance the nutritional value of each protein by compensating for nutritional imbalances or deficiencies [16, 21, 22]. Furthermore, combined protein sources are cost‐effective because they could typically substitute higher levels of FM compared to a single protein in fish feeds [23, 24]. In particular, Kikuchi [24] reported that olive flounder fed diets substituting 35% of FM protein with a combination of 30% soybean meal and 10% blood meal spray dehydrate or 10% CGM showed slightly but not statistically different, greater weight gain (WG) compared to fish fed a diet substituting 35% of FM protein with single soybean meal. However, these low‐FM diets produced comparable WG to that of fish fed a 75% FM‐based diet.

Nevertheless, a high level of substitution with a replacer in feeds often results in deteriorated palatability and reduced feed intake by fish, which consequently results in poor growth performance of fish [16, 25]. Hence, an incorporation of a feed enhancer (stimulant) or a protein feed ingredient exhibiting strong feed attractant activity to a target fish into the low‐FM diets could adequately enhance their palatability and feed consumption (FC) of fish [25–29]. In our recent study [30], we found that among 18 protein feed ingredients, red sea bream showed the strongest attractiveness to jack mackerel meal (JMM), and dietary incorporation of 24% JMM instead of FM statistically enhanced their FC and improved growth performance. In addition to the growth‐enhancing effects of JMM, the biochemical compounds, such as glutamic acid (Glu), His, inosine monophosphate, and/or lactic acid found in jack mackerel muscle extracts, have been shown to induce stimulatory responses in greater amberjack (*Seriola dumerili*) [31], olive flounder [32], and yellowtail (*Seriola quinqueradiata*) [33]. Therefore, manipulating JMM in low‐FM diets can enhance FC and growth in red sea bream, providing a highly sustainable approach to advancing aquaculture techniques.

Red sea bream is considered one of the most economically important marine fish species in the Republic of Korea (hereafter, Korea), along with olive flounder and rockfish. Its production reached 6474 metric tons, accounting for ~8% of the total aquaculture production (81,911 metric tons), and the value of this species was known to be USD 10.4 per kg of fish in Korea in 2024 [34], indicating that red sea bream holds considerable economic value. Due to its economic significance and the sustainable aquaculture industry, exploring sustainable alternatives for FM in the diets of red sea bream has become a critical area of research. As a result, a variety of studies have substituted FM with alternatives [7, 13, 35, 36] and manipulated protein ingredients like JMM [30] and fish soluble, squid meal, and krill meal [29] to improve FC of the red sea bream diets.

Given the growing interest in sustainable dietary substitutes for FM and the challenges faced by the red sea bream aquaculture industry in Korea, this experiment aims to assess the use of combined MM and CGM (MC) as a FM substitute. It also examines the JMM incorporation in low‐FM feeds to assess the growth and feed utilization of red sea bream and economic analysis.

## 2. Materials and Methods

### 2.1. Animal Ethics Statement

All experimental procedures were conducted in accordance with ethical regulations and were approved by the Institutional Animal Care and Use Committee (IACUC) of Korea Maritime and Ocean University (Busan Metropolitan City, Korea) (KMOU IACUC 2021‐04).

### 2.2. Fish Rearing

Healthy red sea bream juveniles of similar size were bought from a private hatchery (Tongyeong‐si, Gyeongsangnam‐do, Korea) and then acclimated for 2 weeks in a 5‐ton circular flow‐through tank (water volume: 3.5 ton) to minimize stress before the experiment. During the acclimatization period, fish were supplied with a commercial extruded pellet (Suhyup Feed, Uiryeong‐gun, Gyeongsang‐do, Korea) with 50% CP and 13% crude lipid (CL) twice daily. After the acclimatization period, 375 juvenile (2.0 ± 0.02 g; mean ± SE) fish were randomly placed to ensure even distribution across treatments into 15 50‐L rectangular flow‐through tanks (water volume: 40 L) (25 fish/tank). The number of fish per tank was chosen based on Dossou et al. [37] and Sim et al. [13] and the optimal stocking density for juvenile red sea bream. Each tank was provided with a mixture of underground seawater and sand‐filtered seawater in a 1:1 ratio at a rate of 4.5 L/min. Water quality was monitored using a digital multimeter (AZ‐86031; AZ Instrument Corp., Taichung, Taiwan) after morning feeding and recorded as follows (mean ± SD): water temperature: 20.1 ± 1.73°C, salinity: 30.7 ± 0.38 g/L, dissolved oxygen: 7.7 ± 0.23 mg/L, and pH: 7.6 ± 0.10. Proper aeration was continuously supplied to each tank, and the photoperiod followed the natural conditions. All tank bottoms were siphon‐cleaned daily. Dead fish were immediately removed upon observation. Additional environmental enrichment was not provided in the experimental tanks. No additional steps to control (Con) confounders were implemented.

### 2.3. Preparation of the Experimental Feeds

A two‐way (FM substitution level [SL] [25% and 50%] × JMM inclusion [without and with]) ANOVA experimental design was adopted. The MC consists of the combined MM (Daekyung Oil and Transportation Co., Ltd., Busan Metropolitan City, Korea) and CGM (Hyunjin Livestock Distribution Co., Ltd., Incheon Metropolitan City, Korea) at a 1:1 ratio, and all ingredients were obtained from local commercial suppliers. Five isoproteic (52.0%) and isolipidic (15.0%) diets were formulated (Table 1), which fulfilled the nutritional requirements of red sea bream [39]. In the Con diet, 60% FM and 13.5% defatted soybean meal as the main protein ingredients, 14.5% wheat flour as the carbohydrate ingredient, and 7.0% fish oil and 2.5% soybean oil as the lipid ingredients were included. In the Con diet, 25% and 50% of FM were replaced with MC, named as the MC25 and MC50 diets, respectively. Additionally, all MC‐substituted diets included 24% JMM at the expense of FM, which is the most optimal inclusion level for red sea bream [30], named as the MC25J and MC50J diets, respectively. Each dietary treatment was tested in triplicate.

**Table 1:** Ingredient and proximate composition of the experimental diets (%, DM basis).

	Experimental diets				
	Con	MC25	MC50	MC25J	MC50J
*Ingredients* (*%*, *DM*)					
Fish meal^1^	60.0	45.0	30.0	21.0	6.0
Combined meat meal and corn gluten meal (MC)^2^	—	13.8	27.6	13.8	27.6
Jack mackerel meal^3^	—	—	—	24.0	24.0
Defatted soybean meal	13.5	13.5	13.5	13.5	13.5
Wheat flour	14.5	15.4	16.3	15.8	16.7
Fish oil	7.0	7.0	7.0	7.0	7.0
Soybean oil	2.5	2.8	3.1	2.4	2.7
Vitamin premix^4^	1.0	1.0	1.0	1.0	1.0
Mineral premix^5^	1.0	1.0	1.0	1.0	1.0
Choline	0.5	0.5	0.5	0.5	0.5
*Nutrients* (*%*, *DM*)					
Dry matter	97.1	97.2	97.2	97.1	97.1
Crude protein (CP)	52.1	51.9	51.8	52.0	52.1
Crude lipid (CL)	15.1	15.1	15.0	15.3	15.3
Ash	11.2	10.5	8.7	10.2	8.3
Carbohydrate^6^	21.6	22.5	24.5	22.5	24.3
Estimated gross energy (kcal/g)^7^	4.3	4.3	4.4	4.4	4.4
Carbohydrate gross energy (CGE, %)^8^	20.1	20.8	22.3	20.7	21.9
Lipid gross energy (LGE, %)^9^	31.6	31.3	30.7	31.6	31.1
CGE/LGE	0.64	0.66	0.73	0.65	0.71

^1^Fish meal (CP: 72.2%, CL: 8.1%, and ash: 15.1%) was imported from Chile (USD 2.41/kg fish meal, USD 1 = 1330 KRW).

^2^Combined meat meal and corn gluten meal (CP: 77.0%, CL: 6.5%, and ash: 5.7%) was the combination of meat meal (CP: 82.9%, CL: 12.1%, and ash: 8.8%; USD 1.05/kg meat meal) and corn gluten meal (CP: 71.1%, CL: 0.9%, and ash: 2.5%; USD 0.75/kg corn gluten meal) at the 1:1 ratio.

^3^Jack mackerel meal (JMM; CP: 73.0%, CL: 9.8%, and ash: 13.6%) was imported from Chile (USD 2.82/kg JMM).

^4^Vitamin premix (g/kg mix): L‐ascorbic acid, 121.2; DL‐α‐tocopheryl acetate, 18.8; thiamine hydrochloride, 2.7; riboflavin, 9.1; pyridoxine hydrochloride, 1.8; niacin, 36.4; Ca‐D‐pantothenate, 12.7; myo‐inositol, 181.8; D‐biotin, 0.27; folic acid, 0.68; p‐aminobenzoic acid, 18.2; menadione, 1.8; retinyl acetate, 0.73; cholecalciferol, 0.003; cyanocobalamin, 0.003.

^5^Mineral premix (g/kg mix): MgSO_4_·7H_2_O, 80.0; NaH_2_PO_4_·2H_2_O, 370.0; KCl, 130.0; ferric citrate, 40.0; ZnSO_4_·7H_2_O, 20.0; Ca‐lactate, 356.5; CuCl, 0.2; AlCl_3_·6H_2_O, 0.15; KI, 0.15; Na_2_Se_2_O_3_, 0.01; MnSO_4_·H_2_O, 2.0; CoCl_2_·6H_2_O, 1.0.

^6^Carbohydrate was calculated by the difference 100–(CP + CL + ash).

^7^Estimated gross energy (kcal/g) was calculated as 4 kcal/g for CP and carbohydrate and 9 kcal/g for CL [38].

^8^Carbohydrate gross energy (CGE, %) was calculated by carbohydrate content (g) × 4 (kacl/g) × 100/estimated gross energy (kcal/g).

^9^Lipid gross energy (LGE, %) was calculated by lipid content (g) × 9 (kacl/g) × 100/estimated gross energy (kcal/g).

The ingredients of the experimental feeds were thoroughly mixed with water at a 3:1 ratio using a vertical mixer (B20GA; Eben Commerce Korea, Ansan‐si, Gyeonggi‐do, Korea). The mixture was pelletized using a pelletizing machine (SMC‐32; SL Company, Incheon Metropolitan City, Korea) with a diameter of 3 mm. The experimental feeds were dried using a drying machine (JW‐1350ED; Jinwoo Electronics Co., Ltd., Hwaseong‐si, Gyeonggi‐do, Korea) at 45°C for 24 h and preserved in a freezer at –20°C until use. The experimental feeds were carefully hand‐fed twice daily (08:30 and 17:30) to minimize uneaten feed for 8 weeks until fish were spontaneously satiated. The amount of diet supplied was recorded daily for each tank after the second feeding, but uneaten diets were not collected.

### 2.4. Handling and Euthanasia of Red Sea Bream

Humane endpoints established for this study included weekly scoring of indicators, including significant increases in mortality, reduced FC, and morphological changes during the feeding period. Euthanasia was determined if the total score of these indicators reached six points or more or if cumulative mortality exceeded 30%. However, no such signs requiring humane endpoints were observed in this study.

After the 8‐week feeding period, all surviving fish were fasted for 24 h and subsequently anesthetized with 50 mg/L tricaine methanesulfonate (MS‐222) for the assessment of growth performance, biological indices, blood chemistry, and biochemical composition. Fish were sampled for biological indices and blood chemistry, and the remaining fish in each tank were euthanized under anesthesia with 50 mg/L MS‐222 by freezing at −20°C and subsequently used for biochemical composition analysis.

### 2.5. Assessment of Growth and Biological Indices of Fish

The surviving fish in each tank were counted and weighed collectively to measure survival, WG, and specific growth rate (SGR). To assess the condition factor (CF), viscerosomatic index (VSI), and hepatosomatic index (HSI), 10 anesthetized fish were randomly chosen from each tank. The growth, feed utilization, and biological indices of fish were determined as the same method in Sim and Cho’s [7] study.

### 2.6. Assessment of Blood Chemistry of Fish

Following the measurement of CF in individual fish, blood samples were taken from the caudal veins of five anesthetized fish per tank using heparinized syringes. The samples were centrifuged at 2700 × *g* for 10 min at 4°C, and the resulting plasma was stored at −70°C until further analysis. Aspartate aminotransferase (AST), alanine aminotransferase (ALT), alkaline phosphatase (ALP), total bilirubin (TBL), total cholesterol (TCO), triglycerides (TRGs), total protein (TPT), and ALB were evaluated using an automatic dry‐chemistry analyzer (FUJI DRI‐CHEM NX500i; Fujifilm Corp., Tokyo, Japan).

Blood was drawn from the caudal vein of five anesthetized fish per tank using syringes. The samples were centrifuged at 2700 × *g* for 10 min at 4°C, and the resulting serum was kept at −70°C until further analysis. Serum lysozyme (LYZ) activity was assessed by turbidity measurement following the method of Lange et al. [40]. A suspension was prepared by dissolving 1.9 mL of *Micrococcus lysodeikticus* (Sigma–Aldrich, St. Louis, MO, USA) in 0.05 M phosphate‐buffered saline at pH 6.2. A 100 µL sample consisting of 95 µL suspension and 5 µL serum sample was incubated for 5 min at 25°C. Absorbance measurements were performed using a microplate reader (Infinite 200 PRO; Tecan Group Ltd., Männedorf, Canton of Zürich, Switzerland) at 530 nm at 0, 15, 30, 45, and 60 min. LYZ activity was measured based on the amount of enzyme needed to decrease the absorbance by 0.001/min. A serum superoxide dismutase (SOD) ELISA kit (MBS705758; MyBiosource Inc., San Diego, CA, USA) precoated with a SOD‐specific antibody was used for the SOD assay. The kit utilized a competitive inhibition enzyme immunoassay technique, with color development inversely proportional to the SOD concentration in the sample. The concentration was determined by creating a standard curve. Absorbance was measured at 450 nm using the same machine as in the LYZ analysis.

### 2.7. Assessment of Biochemical Composition of the Dry and Wet Samples

The dry samples (the sources [FM, JMM, and MC] and all experimental feeds) and the wet samples (10 juvenile fish at the beginning of the feeding experiment and all surviving fish in each tank) were homogenized and used for biochemical composition analysis. The proximate composition, AA profiles, and fatty acid (FA) profiles of all samples were analyzed as the same methods in Sim and Cho’s [7] study.

### 2.8. Economic Analysis of the Study

The economic analysis of the experimental feeds was performed using the following formulas from Martínez‐Llorens et al. [41]. The cost of each experimental feed was determined based on its ingredient composition and the market price of each component. The price of ingredients was as follows: USD 2.41/kg FM, USD 0.90/kg MC, USD 2.82/kg JMM, USD 0.64/kg defatted soybean meal, USD 0.50/kg wheat flour, USD 2.55/kg fish oil, USD 1.65/kg soybean oil, USD 7.67/kg vitamin premix, USD 6.16/kg mineral premix, and USD 1.32/kg choline. The price of red sea bream juveniles was determined at USD 100/kg fish (1 USD per 10 g fish).

### 2.9. Statistical Analysis

All data were assessed using IBM SPSS Statistics for Windows, version 24.0 (IBM Corp., Armonk, NY, USA). Prior to statistical analysis, all parameter means were evaluated for the homogeneity of variances (Levene’s test) and normality (Shapiro–Wilk) tests, and no violations were found (*p* > 0.05). Prior to statistical analysis, percentage data were converted to arcsine‐transformed. A two‐way ANOVA was used to evaluate the effects of FMSL, JMM inclusion, and their interaction (FMSL × JMM inclusion) at a statistical level of *p* < 0.05. Furthermore, the means of the dietary treatments, including the Con diet, were compared using one‐way ANOVA. If statistical differences (*p* < 0.05) were observed among dietary treatments, post hoc analysis was performed using Duncan’s multiple range test [42]. Principal component analysis (PCA) was conducted to identify patterns or major contributing factors among the FA profiles of the whole‐body fish using IBM SPSS Statistics. Average linkage hierarchical clustering was performed to classify each dietary treatment using Pearson correlation after log_2_ transformation of the data.

## 3. Results

### 3.1. AA and FA Profiles of the Sources and the Experimental Diets

MC had comparatively higher leucine (Leu) and phenylalanine (Phe) content among the IAA and the dispensable AA (DAA), except for aspartic acid (Asp) and tyrosine (Tyr) compared to FM (Table S1). JMM had comparatively higher His among the IAA and glycine (Gly) among the DAA compared to FM. The content of Leu and Phe and all DAA, except for Asp and Tyr, tended to increase with elevated dietary replacement levels of FM with MC. The content of His and Gly tended to increase at the same FMSL with JMM inclusion.

MC and JMM contained higher levels of total monounsaturated FA (∑MUFA) compared to FM, whereas total saturated FA (∑SFA) and n–3 highly unsaturated FA (∑n–3 HUFA) were lower (Table S2). The ∑MUFA showed an increasing trend with increased dietary SLs of MC for FM, but ∑SFA and ∑n–3 HUFA tended to decrease. The ∑MUFA showed an increasing trend at the same FMSL with JMM inclusion but decrease for ∑SFA and ∑n–3 HUFA.

### 3.2. Survival and Growth Performance of Red Sea Bream

Dietary FMSL or JMM inclusion had no significant (*p* > 0.6 for both) impact on survival of fish, which ranged from 96.0% to 98.7% (Table 2). Fish fed the diets with 25% substitution of FM (8.7 g/fish and 2.99%/day) showed statistically (*p* < 0.004 and *p* < 0.003, respectively) greater WG and SGR compared with fish fed the diets with 50% substitution of FM (8.4 g/fish and 2.92%/day). In addition, the MC‐substituted diets with JMM inclusion (8.7 g/fish and 2.99%/day) attained statistically (*p* < 0.003 and *p* < 0.002, respectively) greater WG and SGR of fish than those without JMM inclusion (8.4 g/fish and 2.92%/day). In accordance with the multiple comparisons test, WG and SGR of fish fed the MC25J diet were statistically (*p* < 0.02 and *p* < 0.006, respectively) greater than those of fish fed the Con and MC50 diets but did not differ statistically (*p* > 0.05) from those of fish fed the MC25 and MC50J diets. Moreover, no significant differences (*p* > 0.05) were observed in WG and SGR between fish fed Con diet and those fed the MC25, MC50, and MC50J diets.

**Table 2:** Survival (%), weight gain (WG, g/fish), specific growth rate (SGR, %/day), feed consumption (FC, g/fish), feed efficiency (FE), protein efficiency ratio (PER), protein retention (PR, %), condition factor (CF, g/cm^3^), viscerosomatic index (VSI, %), and hepatosomatic index (HSI, %) of red sea bream fed the experimental diets for 8 weeks.

Experimental diets	IW (g/fish)	FW (g/fish)	Survival (%)	WG (g/fish)	SGR^1^ (%/day)	FC (g/fish)	FE^2^	PER^3^	PR^4^ (%)	CF^5^ (g/cm^3^)	VSI^6^ (%)	HSI^7^ (%)
Con	2.0 ± 0.03	10.4 ± 0.19	97.3 ± 1.33	8.4 ± 0.16^bc^	2.93 ± 0.009^bc^	8.07 ± 0.183^bc^	1.04 ± 0.013	2.00 ± 0.026	32.17 ± 0.650	1.88 ± 0.017	6.53 ± 0.024	1.19 ± 0.038
MC25	2.0 ± 0.05	10.5 ± 0.18	97.3 ± 1.33	8.5 ± 0.16^ab^	2.97 ± 0.034^ab^	8.34 ± 0.102^abc^	1.02 ± 0.007	1.98 ± 0.014	33.37 ± 0.404	1.88 ± 0.023	6.58 ± 0.042	1.24 ± 0.069
MC50	2.1 ± 0.05	10.3 ± 0.14	98.7 ± 1.33	8.2 ± 0.09^c^	2.86 ± 0.017^c^	8.01 ± 0.014^c^	1.03 ± 0.010	1.97 ± 0.018	33.34 ± 0.804	1.85 ± 0.029	6.62 ± 0.012	1.31 ± 0.056
MC25J	2.0 ± 0.00	10.8 ± 0.08	98.7 ± 1.33	8.8 ± 0.08^a^	3.01 ± 0.013^a^	8.62 ± 0.030^a^	1.02 ± 0.008	1.96 ± 0.015	32.28 ± 0.737	1.90 ± 0.021	6.54 ± 0.021	1.23 ± 0.060
MC50J	2.0 ± 0.05	10.6 ± 0.08	96.0 ± 2.31	8.6 ± 0.04^ab^	2.98 ± 0.030^ab^	8.39 ± 0.117^ab^	1.02 ± 0.012	1.96 ± 0.022	32.25 ± 0.553	1.87 ± 0.015	6.59 ± 0.012	1.26 ± 0.038
*p*‐Value	—	—	*p* > 0.7	*p* < 0.02	*p* < 0.006	*p* < 0.02	*p* > 0.5	*p* > 0.6	*p* > 0.6	*p* > 0.4	*p* > 0.7	*p* > 0.5
Main effect: FMSL												
25%	—	—	98.0 ± 0.09	8.7 ± 0.10^A^	2.99 ± 0.019^A^	8.48 ± 0.079^A^	1.02 ± 0.005	1.97 ± 0.010	32.83 ± 0.447	1.89 ± 0.015	6.56 ± 0.023	1.23 ± 0.041
50%	—	—	97.3 ± 1.33	8.4 ± 0.09^B^	2.92 ± 0.030^B^	8.20 ± 0.100^B^	1.03 ± 0.007	1.97 ± 0.015	32.79 ± 0.500	1.86 ± 0.015	6.61 ± 0.010	1.29 ± 0.032
Main effect: JMM inclusion												
Without	—	—	98.0 ± 0.09	8.4 ± 0.11^B^	2.92 ± 0.029^B^	8.18 ± 0.086^B^	1.03 ± 0.005	1.97 ± 0.010	33.35 ± 0.402	1.87 ± 0.018	6.60 ± 0.021	1.27 ± 0.043
With	—	—	97.3 ± 1.33	8.7 ± 0.06^A^	2.99 ± 0.016^A^	8.51 ± 0.074^A^	1.02 ± 0.007	1.96 ± 0.012	32.26 ± 0.412	1.89 ± 0.013	6.57 ± 0.015	1.25 ± 0.033
Two‐way ANOVA												
FMSL	—	—	*p* > 0.6	*p* < 0.004	*p* < 0.003	*p* < 0.008	*p* > 0.6	*p* > 0.9	*p* > 0.9	*p* > 0.2	*p* > 0.1	*p* > 0.3
JMM inclusion	—	—	*p* > 0.6	*p* < 0.003	*p* < 0.002	*p* < 0.003	*p* > 0.5	*p* > 0.6	*p* > 0.1	*p* > 0.4	*p* > 0.9	*p* > 0.6
Interaction	—	—	*p* > 0.2	*p* > 0.4	*p* > 0.1	*p* > 0.5	*p* > 0.9	*p* > 0.7	*p* > 0.9	*p* > 0.8	*p* > 0.9	*p* > 0.7

*Note:* Con, the 60% fish meal (FM)–based diet; MC25, dietary 25% substitution of FM with combined meat meal and corn gluten meal (MC); MC50, dietary 50% substitution of FM with MC; MC25J, MC25 with jack mackerel meal (JMM) inclusion; MC50J, MC50 with JMM inclusion. Values are expressed as means of triplicates ± SE. Significant differences (*p* < 0.05) were identified using Duncan’s multiple range test (lowercase letters) and two‐way ANOVA (uppercase letters).

Abbreviations: FMSL, FM substitution level; FW, final weight; IW, initial weight.

^1^Specific growth rate (SGR, %/day) = [Ln final weight of fish (g)–Ln initial weight of fish (g)] × 100/days of the feeding trial].

^2^Feed efficiency (FE) = [total final weight of fish (g)−total initial weight of fish (g) + total weight of dead fish (g)]/total feed consumption (g).

^3^Protein efficiency ratio (PER) = weight gain of fish (g)/protein consumption of fish(g).

^4^Protein retention (PR, %) = protein gain of fish (g) × 100/protein consumption of fish (g).

^5^Condition factor (CF, g/cm^3^) = body weight of fish (g) × 100/total length of fish (cm)^3^.

^6^Viscerosomatic index (VSI, %) = viscera weight of fish (g) × 100/body weight of fish (g).

^7^Hepatosomatic index (HSI, %) = liver weight of fish (g) × 100/body weight of fish (g).

### 3.3. FC, Feed Utilization, and Biological Indices of Fish

Fish fed the diets with 25% substitution of FM exhibited a statistically (*p* < 0.008) higher FC (8.48 g/fish) than those fed the diets with 50% substitution of FM (8.20 g/fish). Moreover, fish fed the MC‐substituted diets with JMM inclusion showed a statistically (*p* < 0.003) higher FC (8.51 g/fish) than those fed the MC‐substituted diets without JMM inclusion (8.18 g/fish). In the multiple comparison test, FC of fish fed the MC25J diet was statistically (*p* < 0.02) higher than that of fish fed the Con and MC50 diets but did not differ statistically (*p* > 0.05) from that of fish fed the MC25 and MC50J diets. Moreover, no significant difference (*p* > 0.05) was obtained in FC between fish fed Con diet and those fed the MC25, MC50, and MC50J diets.

Feed efficiency (FE) (1.02–1.04), protein efficiency ratio (PER) (1.96–2.00), and protein retention (PR) (32.17%–33.37%) were statistically unaffected by dietary FMSL (*p* > 0.6, *p* > 0.9, and *p* > 0.9, respectively) or JMM inclusion (*p* > 0.5, *p* > 0.6, and *p* > 0.1, respectively).

CF (1.85–1.90 g/cm^3^), VSI (6.53%–6.62%), and HSI (1.19%–1.31%) were not statistically affected by dietary FMSL (*p* > 0.2, *p* > 0.1, and *p* > 0.3, respectively) or JMM inclusion (*p* > 0.4, *p* > 0.9, and *p* > 0.6, respectively).

### 3.4. Blood Chemistry of Fish

Plasma AST varied from 53.8 to 56.8 U/L, ALT varied from 8.4 to 8.7 U/L, ALP varied from 200.2 to 219.8 U/L, TBL was 1.0 mg/dL, TCO varied from 243.1 to 257.8 mg/dL, TRG varied from 212.0 to 232.1 mg/dL, TPT varied from 3.5 to 3.8 g/dL, and ALB varied from 0.9 to 1.0 g/dL (Table 3). Serum LYZ activity varied from 80.5 to 103.3 U/mL, and SOD varied from 1.7 to 1.9 ng/mL. Neither the plasma nor the serum parameters of fish were statistically (*p* > 0.05 for all) influenced by dietary FMSL or JMM inclusion.

**Table 3:** Blood chemistry of red sea bream fed the experimental diets for 8 weeks.

Experimental diets	Plasma parameters								Serum parameters	
	AST (U/L)	ALT (U/L)	ALP (U/L)	TBL (mg/dL)	TCO (mg/dL)	TRG (mg/dL)	TPT (g/dL)	ALB (g/dL)	LYZ activity (U/mL)	SOD (ng/mL)
Con	55.4 ± 2.19	8.7 ± 0.19	200.2 ± 1.42	1.0 ± 0.14	249.1 ± 4.04	213.0 ± 9.43	3.6 ± 0.04	1.0 ± 0.06	80.5 ± 12.5	1.7 ± 0.07
MC25	56.8 ± 2.28	8.7 ± 0.69	209.0 ± 11.32	1.0 ± 0.15	257.8 ± 7.10	212.0 ± 5.40	3.8 ± 0.08	0.9 ± 0.12	102.8 ± 15.7	1.7 ± 0.04
MC50	55.7 ± 1.35	8.4 ± 0.40	219.8 ± 8.72	1.0 ± 0.09	251.8 ± 4.80	220.0 ± 9.13	3.5 ± 0.11	0.9 ± 0.13	99.3 ± 7.8	1.9 ± 0.18
MC25J	53.8 ± 1.57	8.6 ± 0.44	210.1 ± 6.30	1.0 ± 0.04	243.1 ± 8.68	228.7 ± 3.56	3.8 ± 0.18	1.0 ± 0.06	90.3 ± 12.6	1.8 ± 0.14
MC50J	54.9 ± 1.44	8.6 ± 0.59	213.3 ± 11.56	1.0 ± 0.14	253.0 ± 8.57	232.1 ± 8.92	3.8 ± 0.14	0.9 ± 0.07	103.3 ± 15.1	1.9 ± 0.16
*p*‐Value	*p* > 0.8	*p* > 0.9	*p* > 0.6	*p* > 0.6	*p* > 0.9	*p* > 0.3	*p* > 0.2	*p* > 0.8	*p* > 0.6	*p* > 0.8
Main effect: FMSL										
25%	55.3 ± 1.41	8.6 ± 0.37	209.6 ± 5.80	1.0 ± 0.07	250.4 ± 5.99	220.3 ± 4.72	3.8 ± 0.09	0.9 ± 0.06	96.6 ± 9.41	1.7 ± 0.07
50%	55.3 ± 0.90	8.5 ± 0.32	216.6 ± 6.63	1.0 ± 0.08	252.4 ± 4.40	226.1 ± 6.32	3.6 ± 0.10	0.9 ± 0.07	101.3 ± 7.65	1.9 ± 0.11
Main effect: JMM inclusion										
Without	56.2 ± 1.21	8.6 ± 0.36	214.4 ± 6.83	1.0 ± 0.08	254.8 ± 4.06	216.0 ± 5.07	3.6 ± 0.10	0.9 ± 0.08	101.1 ± 7.87	1.8 ± 0.09
With	54.3 ± 0.99	8.6 ± 0.33	211.7 ± 5.93	1.0 ± 0.07	248.1 ± 5.88	230.4 ± 4.37	3.8 ± 0.10	0.9 ± 0.05	96.8 ± 9.25	1.8 ± 0.10
Two‐way ANOVA										
FMSL	*p* > 0.9	*p* > 0.8	*p* > 0.4	*p* > 0.8	*p* > 0.7	*p* > 0.4	*p* > 0.6	*p* > 0.8	*p* > 0.7	*p* > 0.4
JMM inclusion	*p* > 0.2	*p* > 0.9	*p* > 0.7	*p* > 0.8	*p* > 0.3	*p* > 0.07	*p* > 0.3	*p* > 0.8	*p* > 0.7	*p* > 0.7
Interaction	*p* > 0.5	*p* > 0.8	*p* > 0.7	*p* > 0.8	*p* > 0.3	*p* > 0.7	*p* > 0.3	*p* > 0.4	*p* > 0.5	*p* > 0.8

*Note:* Con, the 60% fish meal (FM)–based diet; MC25, dietary 25% substitution of FM with combined meat meal and corn gluten meal (MC); MC50, dietary 50% substitution of FM with MC; MC25J, MC25 with jack mackerel meal (JMM) inclusion; MC50J, MC50 with JMM inclusion. Values are expressed as means of triplicates ± SE.

Abbreviations: ALB, albumin; ALP, alkaline phosphatase; ALT, alanine aminotransferase; AST, aspartate aminotransferase; FMSL, FM substitution level; LYZ activity, lysozyme activity; SOD, superoxide dismutase; TBL, total bilirubin; TCO, total cholesterol; TPT, total protein; TRGs, triglycerides.

### 3.5. Biochemical Composition of the Whole‐Body Fish

Moisture content of the whole‐body fish varied from 69.0% to 71.1%, CP content varied from 16.4% to 17.1%, CL content varied from 7.3% to 7.8%, and ash content varied from 4.0% to 4.4% (Table 4). The proximate composition of the whole‐body fish was statistically (*p* > 0.05 for all) unaffected by dietary FMSL or JMM inclusion.

**Table 4:** Proximate composition (% of wet weight) of the whole‐body red sea bream fed the experimental diets for 8 weeks.

Experimental diets	Moisture	Crude protein	Crude lipid	Ash
Con	69.8 ± 0.41	16.4 ± 0.10	7.3 ± 0.23	4.4 ± 0.25
MC25	70.6 ± 0.50	17.1 ± 0.19	7.5 ± 0.20	4.3 ± 0.10
MC50	69.0 ± 0.75	17.0 ± 0.23	7.8 ± 0.29	4.0 ± 0.15
MC25J	71.1 ± 0.17	16.8 ± 0.31	7.4 ± 0.00	4.1 ± 0.21
MC50J	70.5 ± 0.53	16.7 ± 0.10	7.7 ± 0.18	4.0 ± 0.06
*p*‐Value	*p* > 0.1	*p* > 0.2	*p* > 0.6	*p* > 0.4
Main effect: FMSL				
25%	70.8 ± 0.26	16.9 ± 0.17	7.5 ± 0.09	4.2 ± 0.11
50%	69.7 ± 0.52	16.8 ± 0.13	7.7 ± 0.15	4.0 ± 0.07
Main effect: JMM inclusion				
Without	69.8 ± 0.53	17.0 ± 0.13	7.6 ± 0.17	4.2 ± 0.10
With	70.3 ± 0.29	16.7 ± 0.15	7.5 ± 0.09	4.1 ± 0.10
Two‐way ANOVA				
FMSL	*p* > 0.06	*p* > 0.6	*p* > 0.2	*p* > 0.2
JMM inclusion	*p* > 0.09	*p* > 0.2	*p* > 0.6	*p* > 0.4
Interaction	*p* > 0.4	*p* > 0.9	*p* > 0.8	*p* > 0.6

*Note:* Con, the 60% fish meal (FM)–based diet; MC25, dietary 25% substitution of FM with combined meat meal and corn gluten meal (MC); MC50, dietary 50% substitution of FM with MC; MC25J, MC25 with jack mackerel meal (JMM) inclusion; MC50J, MC50 with JMM inclusion. Values are expressed as means of triplicates ± SE.

Abbreviation: FMSL, FM substitution level.

The AA profiles of the whole‐body fish were statistically (*p* > 0.05 for all) unchanged by dietary FMSL or JMM inclusion (Table S3).

**Figure 1:**
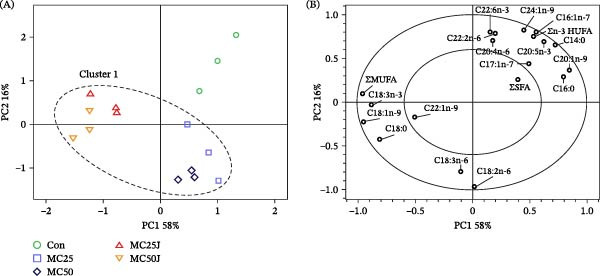
Principal component analysis score plot (A) and correlation loading plot (B) for the fatty acid profiles of the whole‐body red sea bream fed the experimental diets. PC1: first principal component; PC2: two principal component; Con: the 60% fish meal (FM)–based diet; MC25: dietary 25% substitution of FM with combined meat meal and corn gluten meal (MC); MC50: dietary 50% substitution of FM with MC; MC25J: MC25 with jack mackerel meal (JMM) inclusion; MC50J: MC50 with JMM inclusion; ∑SFA: total saturated fatty acids; ∑MUFA: total monounsaturated fatty acids; ∑n–3 HUFA: total n–3 highly unsaturated fatty acids.

PCA of the whole‐body FA profiles showed one dominant cluster of four treatments (the MC25, MC50, MC25J, and MC50J diets), with one treatment (the Con diet) clearly separated from this group (Figure 1A). The first two principal components (PC1, 58% and PC2, 16%) explained 74% of the total variation. The eicosenoic acid (C20:1n–9), palmitic acid (C16:0), myristic acid (C14:0), and eicosapentaenoic acid (EPA, C20:5n–3) were identified as the most significant variables in PC1, and myristic acid, tetracosenoic acid (C24:1n–9), docosahexaenoic acid (DHA, C22:6n–3), palmitoleic acid (C16:1n–7), ∑n–3 HUFA, docosadienoic acid (C22:2n–6), and arachidonic acid (C20:4n–6) were identified as the most significant variables in PC2 (Figure 1B). In addition, the ∑n–3 HUFA, EPA, DHA, docosadienoic acid, eicosenoic acid, myristic acid, palmitic acid, palmitoleic acid, and tetracosenoic acid were highly correlated with the FA profiles of the whole‐body fish fed the Con diet. Linoleic acid (C18:2n–6), γ‐linolenic acid (C18:3n–6), oleic acid (C18:1n–9), and stearic acid (C18:0) were highly correlated with the FA profiles of the whole‐body fish fed the MC25, MC50, MC25J, and MC50J diets.

The ∑SFA of the whole‐body fish was not statistically influenced by dietary FMSL (*p* > 0.9) or JMM inclusion (*p* > 0.4) (Table S4). Furthermore, the ∑MUFA of the whole‐body fish was not statistically (*p* > 0.2) affected by FMSL. However, fish fed the MC‐substituted diets with JMM inclusion showed a statistically (*p* < 0.0001) higher ∑MUFA of the whole‐body fish (34.96% of total FA) than those fed the MC‐substituted diets without JMM inclusion (33.65% of total FA). Moreover, fish fed the diets with 25% substitution of FM exhibited a statistically (*p* < 0.001) higher ∑n–3HUFA of the whole‐body fish (6.78% of total FA) than those fed the diets with 50% substitution of FM (6.51% of total FA). However, the ∑n–3 HUFA of whole‐body fish was statistically (*p* > 0.6) unaffected by dietary JMM inclusion. In the multiple comparison test, fish fed the MC25J and MC50J diets had a statistically higher (*p* < 0.0001) ∑MUFA than that fed the Con, MC25, and MC50 diets. Additionally, fish fed the Con diet had a statistically higher (*p* < 0.0001) ∑n–3 HUFA than that fed the MC25, MC50, MC25J, and MC50J diets.

### 3.6. Economic Analysis of the Study

The Con diet had the highest price (USD 1.97/kg) among the experimental diets (Table 5). The diets with 25% substitution of FM (USD 1.75/kg) exhibited statistically (*p* < 0.0001) higher economic conversion ratio (ECR) compared with the diets with 50% substitution of FM (USD 1.52/kg). Moreover, the MC‐substituted diets with JMM inclusion (USD 1.68/kg) showed statistically (*p* < 0.0001) higher ECR compared with the MC‐substituted diets without JMM inclusion (USD 1.58/kg). In the multiple comparison test, the Con diet showed a statistically (*p* < 0.0001) higher ECR compared with all other diets.

**Table 5:** Economic parameters of the feeding trial.

Experimental diets	Diet price (USD/kg)	ECR^1^ (USD/kg)	EPI^2^ (USD/fish)
Con	1.97	1.89 ± 0.025^a^	1.03 ± 0.018
MC25	1.74	1.70 ± 0.012^c^	1.04 ± 0.018
MC50	1.51	1.47 ± 0.014^e^	1.02 ± 0.014
MC25J	1.83	1.80 ± 0.014^b^	1.06 ± 0.008
MC50J	1.60	1.57 ± 0.018^d^	1.05 ± 0.007
*p*‐Value	—	*p* < 0.0001	*p* > 0.3
Main effect: FMSL			
25%	—	1.75 ± 0.024^A^	1.05 ± 0.025
50%	—	1.52 ± 0.024^B^	1.03 ± 0.009
Main effect: JMM inclusion			
Without	—	1.58 ± 0.127^B^	1.03 ± 0.027
With	—	1.68 ± 0.052^A^	1.05 ± 0.006
Two‐way ANOVA			
FMSL	—	*p* < 0.0001	*p* > 0.2
JMM inclusion	—	*p* < 0.0001	*p* > 0.09
Interaction	—	*p* > 0.9	*p* > 0.9

*Note:* Con, the 60% fish meal (FM)–based diet; MC25, dietary 25% substitution of FM with combined meat meal and corn gluten meal (MC); MC50, dietary 50% substitution of FM with MC; MC25J, MC25 with jack mackerel meal (JMM) inclusion; MC50J, MC50 with JMM inclusion. Values are expressed as means of triplicates ± SE. Significant differences (*p* < 0.05) were identified using Duncan’s multiple range test (lowercase letters) and two‐way ANOVA (uppercase letters).

Abbreviation: FMSL, FM substitution level.

^1^Economic conversion ratio (ECR, USD/kg) = feed consumption of fish (kg/fish)/weight gain (kg/fish) × diet price (USD/kg).

^2^Economic profit index (EPI, USD/fish) = [final weight (kg/fish) × fish sale price (USD/kg)]–[ECR (USD/kg) × weight gain (kg/fish)].

The economic profit index (EPI) was not statistically (*p* > 0.2, and *p* > 0.09, respectively) influenced by dietary FMSL or JMM inclusion.

## 4. Discussion

In general, changes in ingredient composition resulting from replacing FM with a replacer in diets can affect the flavor, leading to deteriorated palatability and reduced fish appetite [20]. The diets with 25% substitution of FM attained superior WG, SGR, and FC of red sea bream compared to the diets with 50% substitution of FM, indicating that the dietary high level of FM replacement with MC led to deteriorated palatability, reduced FC by fish, and ultimately lowered the growth performance of fish in this experiment. Similarly, the high level of FM substitution with a blended scallop by‐product and fermented soybean meal, or with a combination of MM and CGM in feeds, led to deteriorated growth performance of red sea bream and rockfish, respectively, primarily due to reduced FC [43, 44].

To enhance the palatability of low‐FM diets, the inclusion of main feed ingredients exhibiting strong feed enhancer and/or attractant responses to a target fish can typically have a positive effect on the palatability and FC of red sea bream [29, 30] and olive flounder [28]. In particular, incorporating 24% JMM, which showed the strongest attractiveness among 18 feed ingredients, in place of FM in the red sea bream diets resulted in better growth performance and FC compared to a 60% FM‐based diet [30]. Likewise, including 24% JMM in the MC‐substituted diets resulted in better growth performance and FC in fish compared to the MC‐substituted diets without JMM inclusion in this experiment. This might suggest that incorporating JMM in the MC‐substituted diets contributed to improved FC, which was well reflected in the enhanced growth of fish. In particular, Glu, His, inosine monophosphate, and/or lactic acid in the muscle extracts of jack mackerel have been reported to trigger stimulatory responses in greater amberjack, olive flounder, and yellowtail [31–33]. Since these feed stimulants are present in JMM, the inclusion of JMM in the low‐FM feeds enhanced palatability, triggered feeding behavior, and ultimately increased FC in red sea bream. In this experiment, the higher His content in the MC‐substituted diets with JMM inclusion, compared to those without JMM inclusion in the same FMRL, might have stimulated the feeding behavior of red sea bream, ultimately leading to increased FC. However, since the specific compounds in JMM responsible for inducing a strong feeding response in red sea bream were not identified in the current experiment, further research is needed to elucidate this mechanism.

The greatest growth performance of red sea bream was observed in the MC25J diet, which was attributed to the highest FC. Similarly, the highest FC was observed in fish fed a low‐FM diet with the inclusion of a combination of 15% feed ingredients as feed enhancers, which was well reflected in the greatest growth performance when fish were supplied with a 60% FM‐based diet, a diet replacing 60% of FM by soy protein concentrate; the low‐FM diets with the inclusion of 2.5% crystalline AA, 10% each of fish soluble, krill meal, and squid meal; and a combination of 15% feed ingredients (5% each of fish soluble, krill meal, and squid meal) [45]. Furthermore, FC of large yellow croaker (*Larimichthys crocea*) fed a diet in which 75% of FM was replaced with krill meal was statistically higher than that of fish fed a 40% FM‐based diet, and SGR of fish fed the former was slightly, but not statistically, greater than that of fish fed the latter when fish were supplied with a 40% FM‐basal diet or diets replacing graded levels (15%–75%) of FM with krill meal [46]. This might be due to the leaching of smell‐causing chemical compounds from krill meal into the water, which increased the FC of fish [47].

No significant differences in WG, SGR, and FC of fish fed the Con and MC50 diets indicated that MC could substitute for 50% of FM in a 60% FM‐basal diet without statistically reducing the growth and FC in fish in this experiment. In contrast, our studies [7, 13] have reported that the substitutability of FM with the same grade (pet‐grade) MM and CGM as single protein sources is 40% and 20% in the 55% and 60% FM‐based diets, respectively, without negatively influencing the growth performance and FC of red sea bream. Disparities in MM and CGM substitutability for FM arise from the synergistic effect of the combining animal (MM) and plant (CGM) protein sources, which can compensate for the nutritional imbalances or deficiencies of a single source [16, 21, 22]. In general, pet‐grade MM contains lower IAA content, except for Arg content [7], whereas CGM has higher Leu and Phe content compared to FM [13]. However, when these proteins are combined, Leu and Phe content exceed those in FM, which was well reflected in higher Leu and Phe content in the MC‐substituted diets without JMM than the Con diet. This highlights MC’s potential as an effective FM substitute in red sea bream diets due to the synergistic effect.

However, replacing 50% of FM with the combination of MM and CGM at a 1:1 ratio led to inferior growth performance of fingerling red sea bream compared to a 60% FM‐basal feed in the 12‐week feeding experiment [48]. The differences in dietary SLs for FM in the present study (up to 50%) and in the study by Gunathilaka et al. [48] might result from those in the dietary ratio of carbohydrate gross energy (CGE, %) to lipid gross energy (LGE, %). Koshio [49] reported that the dietary CGE/LGE can influence growth performance, with a ratio of lower than 1 being optimal for red sea bream. In the current experiment, the CGE/LGE in the MC50 diet was 0.73 (Table 1), whereas it was 1.01 in the study by Gunathilaka et al. [48]. Therefore, improved growth performance was observed in fish fed the MC50 diet in this experiment compared to those in Gunathilaka et al. [48], as the CGE/LGE is lower than 1. Additionally, factors, such as dietary CP and CL content, the rearing period, and other environmental conditions, might have also contributed to the variations in FMSL with MC in red sea bream diets.

All experimental diets fulfilled the dietary requirements of Arg (2.37% of the diet), Lys (1.79% of the diet), and valine (Val, 0.90% of the diet) for red sea bream, as established by Forster and Ogata [50] and Rahimnejad and Lee [51, 52]. The higher Leu and Phe content in the MC50 and MC50J diets might account for the comparable growth and FE observed in fish fed the Con diet in this experiment. Leu is indispensable for optimal growth performance and maintaining physiological stability in fish [53], while Phe is crucial for normal growth and metabolic functions [54]. For instance, the lowest WG was obtained in Nile tilapia (*Oreochromis niloticus*) fed a diet with the lowest Leu content (0.53% of the diet) when fish were provided with 70% casein‐based diets containing varying levels of Leu (0.53%, 0.81%, 1.09%, 1.32%, 1.56%, and 1.81%) [55]. Moreover, the WG of hybrid tilapia (*O. niloticus* × *O. aureus*) fed a diet with the lowest Phe content (0.43% of the diet) was the lowest, while those fed a diet with the highest Phe content (1.91% of the diet) showed WG comparable to those fed a diet with optimal Phe content (1.30% of the diet) [56]. Although the optimal Leu and Phe content for red sea bream diets is not known yet, their higher content in the MC50 and MC50J diets compared to the Con diet may explain why 50% of FM could be substituted by MC in the red sea bream diets, regardless of JMM inclusion.

The n–3 HUFA is important for the survival and normal growth of carnivorous fish species, such as black sea bream (*Acanthopagrus schlegeli*) and golden pompano (*Trachinotus ovatus*) [57, 58]. The ∑MUFA showed a tendency to increase with higher dietary FMSL with MC, but ∑SFA and ∑n–3 HUFA showed a tendency to decrease, while the ∑MUFA showed an increasing trend at the same FMSL with JMM inclusion, but ∑SFA and ∑n–3 HUFA showed a decreasing trend. However, despite the lower ∑n–3 HUFA in all MC‐substituted diets regardless of JMM inclusion compared to the Con diet, the growth performance of red sea bream fed the formers was comparable to or even superior to fish fed the Con diet. This suggested that dietary ∑n–3 HUFA did not negatively impact the growth performance of red sea bream in the current experiment.

None of the FE, PER, and PR of fish was statistically influenced by dietary FMSL or JMM inclusion in the current experiment, suggesting that the growth of red sea bream was proportionally reflected by FC. Likewise, the highest growth performance and FC were observed in rainbow trout (*Oncorhynchus mykiss*) fed a diet replacing 23% of FM with soybean meal supplemented with 1% betaine, but feed conversion ratio and PER were unaffected by dietary treatments when fish were supplied with a 65% FM‐based diet, a diet replacing 23% of FM with soybean meal supplemented with 1% betaine, or the diets replacing 46% of FM with soybean meal supplemented with or without 1% betaine [59]. Kader et al. [29] also demonstrated that red sea bream fed a 70% dehulled soybean meal–substituted diet with 20% feed ingredients showed the highest growth and FC, but feed utilization was not affected by dietary treatments when fish were supplied with a 60% FM‐based diet or the diets replacing 70%, 80%, 90%, and 100% of FM by dehulled soybean meal with 20% feed ingredients (compromising 10% fish soluble and 5% each of squid meal and krill meal), known as feed enhancers. Similarly, elevating FM replacement with fermented rapeseed meal or JMM in red sea bream diets resulted in a linear decrease or increase, respectively, in growth performance and FC, but feed utilization was unaffected by dietary treatments [30, 36].

CF is an index used to measure the general health status of fish [60], and a low CF value indicates that fish have focused on length growth [61]. VSI reflects the health status [62], and HSI is utilized to assess the impact of feeding on the liver, a key organ in metabolism processes [63]. The biological indices were not affected by dietary FMSL or JMM inclusion in the current study, indicating that up to 50% of FM could be substituted with MC in diets irrespective of JMM inclusion without causing negative impacts on the biological indices of red sea bream. Similarly, dietary FM substitution with single plant (CGM and soy protein concentrate) and animal (MM and chicken by‐product meal) proteins and their combined proteins did not influence CF, VSI, and HSI in red sea bream [48]. Monge‐Ortiz et al. [64] also explained that CF and HSI of *S. dumerili* were unaffected by FM replacement by animal (MM and krill meal) and plant (CGM) protein blends in diets, but VSI was. Furthermore, dietary CGM and fermented rapeseed meal replacement for FM did not change the CF, VSI, and HSI of spotted rose snapper [12] and red sea bream [36], respectively.

Plasma parameters have been widely acknowledged as reliable indicators of fish health [65]. The LYZ serves as an important indicator of innate immunity in fish [66]. SOD is an antioxidant enzyme that plays a vital role in the antioxidant defense mechanism, actively participating in eliminating ROS and safeguarding organisms from oxidative damage [67]. Dietary treatments did not affect either plasma parameters or serum parameters in red sea bream in this experiment, indicating stable physiological status. This suggested that the alternative regimen did not induce stress or metabolic disturbances in red sea bream. Likewise, plasma measurements and serum LYZ activity and SOD of olive flounder were not changed by various inclusion levels of JMM in diets [68]. Similarly, plasma TPT, AST, and ALT were not influenced by FM substitution with blended scallop by‐product and fermented soybean meal in red sea bream diets, but plasma TRG and TCO were [43]. Furthermore, substituting FM with single plant and animal proteins as well as their blended proteins did not affect plasma AST, ALT, TBL, TPT, ALB, and TCO and serum SOD in red sea bream, but it did affect serum LYZ activity [48].

The proximate composition and AA profiles of the whole‐body fish were not influenced by dietary FMSL or JMM inclusion in this experiment. This indicated that MC could substitute up to 50% of FM in the 60% FM‐based diets, regardless of JMM inclusion, without deteriorating the proximate composition and AA profiles of the whole‐body fish. Similarly, the chemical composition of the whole‐body red sea bream was unaffected by the low‐FM diets with 20% feed ingredients, known as feed enhancers [29]. Likewise, FM substitution with a mix of animal (fish residue meal) and plant (soy protein concentrate) proteins in feeds did not affect the moisture, CP, CL, ash, and energy of the whole body of red sea bream [35]. However, dietary 60% FM replacement with the combined animal and plant sources led to lower CP and CL of the whole body of rockfish compared to a 65% FM‐based diet, but AA profiles, except for Gly and proline in the whole‐body fish, were unaffected by dietary FM replacements [44]. Furthermore, the blended animal and plant protein feed ingredient replacement for FM in diets did not alter the moisture, CL, ash, and AA profiles, except for Arg and Gly of the whole‐body *S. dumerili* [64]. Likewise, the proximate composition and AA profiles of the whole body of blunt snout bream (*Megalobrama amblycephala*) were not changed by FM replacement with rice protein concentrate with or without Lys supplementation in diets [69]. This is likely due to the lower rate of muscle protein synthesis in fish compared to the total body protein synthesis rate [70]. Similar to other vertebrates, fish have muscle as the largest component of lean body mass, and muscle protein accounts for about 50% of the body’s TPT. However, its synthesis rate is only ca. 20% of the total body protein synthesis [70]. For this reason, the AA profiles of the whole body of fish might be largely unaffected by dietary FM replacements.

PCA of the whole‐body FA profiles showed one dominant cluster of the MC‐substituted diets, with one treatment (the FM‐basal diet) clearly separated from this group. The ∑n–3 HUFA including DHA and EPA, as well as linoleic acid and oleic acid, was highly correlated with the FA profiles of the whole body of fish fed the Con diet and the MC‐substituted diets, respectively. These differences were primarily associated with the reduced ∑n–3 HUFA levels resulting from dietary FM replacement with MC. Moreover, reduced ∑n–3 HUFA levels in the whole‐body fish might affect fish meat quality, including flavor and nutritional value [71, 72]. Therefore, fish oil supplementation may still be required to optimize the nutritional value and flavor of fish, although growth performance was not affected by dietary n–3 HUFA in this study.

Fish fed the diets with 25% substitution of FM showed higher whole‐body ∑n–3 HUFA than those fed the diets with 50% substitution of FM. This was well reflected from higher ∑n–3 HUFA in the former compared to the latter. The highest ∑n–3 HUFA was observed in fish fed the Con diet in multiple comparisons, likely due to the highest ∑n–3 HUFA in the Con diet. Similarly, increased dietary MM or CGM substitution for FM led to decreased ∑n–3 HUFA in the whole‐body red sea bream [7, 13]. Moreover, increased dietary FM substitution with *Tenebrio molitor* meal linearly decreased ∑n–3 but increased ∑n–6. This change was well reflected in the whole‐body European sea bass (*Dicentrarchus labrax*), showing decreased ∑n–3 but increased ∑n–6 [73]. Hu et al. [18] also showed that increasing the FM replacement with animal protein blends in diets resulted in a linear decrease in ∑n–3 HUFA and lowered ∑n–3 HUFA in the muscle of Asian seabass (*Lateolabrax japonicus*). Likewise, increasing FM substitution with a blend of plant protein sources (corn gluten, peas, soybean meal, and wheat) in diets led to a linear increase in total n–6 polyunsaturated FA, which was clearly reflected in the increased total n–6 polyunsaturated FA in the muscle of Senegalese sole (*Solea senegalensis*) [74].

In general, JMM (USD 2.82/kg) is among the costliest sources of FM. Therefore, incorporating JMM into diets necessitates an economic analysis to assess its viability and cost‐effectiveness. The diet price and ECR tended to decrease with increased MC replacement for FM in diets. The MC25J diet was the highest EPI, although the EPI did not differ statistically among dietary treatments. From an economic perspective, the EPI is a crucial parameter for evaluating dietary profitability for fish farmers, as it considers factors, such as growth performance, FC, diet price, and the fish sale price. Therefore, the MC25J diet appears to offer economic benefits for fish farmers.

These findings demonstrated the effectiveness of MC as a FM replacer in red sea bream diets, supporting the development of a more sustainable and cost‐effective alternative to FM. In addition, the JMM inclusion in low‐FM feeds can improve red sea bream growth, thereby enhancing productivity and advancing aquaculture techniques. Although the 8‐week feeding period was sufficient to evaluate the growth performance, it may not be adequate to fully assess the health status. Therefore, further long‐term, commercial‐scale feeding trials are necessary to assess the feasibility and practicality of substituting MC for FM in red sea bream diets.

## 5. Conclusion

The diets with 25% substitution of FM achieved higher growth performance and FC in red sea bream than those with 50% substitution of FM. Moreover, fish fed the MC‐substituted diets with JMM inclusion exhibited superior growth performance and FC compared to those without JMM inclusion. The MC25J diet appears to represent the best feeding strategy for enhancing the growth performance and FC in red sea bream as well as maximizing economic returns for farmers.

## Author Contributions

**Yu Jin Sim**: conceptualization, methodology, investigation, data curation, writing – original draft. **Sung Hwoan Cho**: conceptualization, data curation, methodology, funding acquisition, writing – review and editing.

## Funding

This work was supported by the National Research Foundation of Korea (NRF) grant funded by the Korea government (MSIT) (Grant RS‐2026‐25477661). This work was supported by the National Research Foundation of Korea grant funded by the Korean government (Grant 2020R1A2C1009903).

## Conflicts of Interest

The authors declare no conflicts of interest.

## Supporting Information

Additional supporting information can be found online in the Supporting Information section.

## Supporting information


**Supporting Information** Table S1: Amino acid (% of the diet) profiles of the main protein ingredients and the experimental diets. Table S2: Fatty acid (% of total fatty acids) profiles of the main protein ingredients and the experimental diets. Table S3: Amino acid (% of wet weight) profiles of the whole‐body red sea bream fed the experimental diets for 8 weeks. Table S4: Fatty acid (% of total fatty acids) profiles of the whole‐body red sea bream fed the experimental diets for 8 weeks.

## Data Availability

The data that support the findings of this study are available from the corresponding author upon reasonable request.

## References

[1] Boyd C. E. , McNevin A. A. , and Davis R. P. , The Contribution of Fisheries and Aquaculture to the Global Protein Supply, Food Security. (2022) 14, no. 3, 805–827, 10.1007/s12571-021-01246-9. 35075379 PMC8771179

[2] Gokulakrishnan M. , Kumar R. , and Ferosekhan S. , et al.Bio-Utilization of Brewery Waste (Brewer’s Spent Yeast) in Global Aquafeed Production and Its Efficiency in Replacing Fishmeal: From a Sustainability Viewpoint, Aquaculture. (2023) 565, 10.1016/j.aquaculture.2022.739161, 739161.

[3] Dai J. , Luo H. , Liu Z. , and Hu Y. , Evaluation of Fish Meal Replacement by *Methylcoccus capsulatus* Protein in Diets for Juvenile Chinese Softshell Turtle (*Pelodiscus sinensis*), Aquaculture. (2024) 587, 10.1016/j.aquaculture.2024.740857, 740857.

[4] Wei X. , Meng S. , and Wang Y. , et al.Effects of Replacing Fish Meal With Biofloc Meal on Growth Performance, Nutrients Metabolism, Immune Response and Intestinal Microbiota of Common Carp (*Cyprinus carpio*), Aquaculture. (2024) 591, 10.1016/j.aquaculture.2024.741124, 741124.

[5] Hicks T. M. and Verbeek C. J. R. , Dhillon G. S. , Meat Industry Protein By-Products: Sources and Characteristics, Protein Byproducts: Transformation From Environmental Burden Into Value-Added Products, 2016, Academic Press, 37–61, 10.1016/B978-0-12-802391-4.00003-3.

[6] Lee Y. and Lee S. , The use of Meat Meal as a Dietary Protein Source Replacing Fish Meal in Juvenile Rockfish *Sebastes schlegeli* , Journal of Aquaculture. (2005) 18, 92–97.

[7] Sim Y. J. and Cho S. H. , Effect of Partial or Complete Substitution of Fish Meal by Meat Meal in Diets on the Growth Performance and Feed Availability of Juvenile Red Sea Bream (*Pagrus major*), Aquaculture Nutrition. (2025) 2025, no. 1, 10.1155/anu/9589317, 9589317. 40182192 PMC11968162

[8] Stone D. A. J. , Allan G. L. , Parkinson S. , and Rowland S. J. , Replacement of Fish Meal in Diets for Australian Silver Perch, *Bidyanus bidyanus*: III. Digestibility and Growth Using Meat Meal Products, Aquaculture. (2000) 186, no. 3-4, 311–326, 10.1016/S0044-8486(99)00381-6.

[9] Lee S.-M. , Apparent Digestibility Coefficients of Various Feed Ingredients for Juvenile and Grower Rockfish (*Sebastes schlegeli*), Aquaculture. (2002) 207, no. 1-2, 79–95, 10.1016/S0044-8486(01)00751-7.

[10] Ha M. S. , Cho S. H. , and Kim T. , Dietary Substitution of Fish Meal by Meat Meal: Effects on Juvenile Olive Flounder (*Paralichthys olivaceus*) Growth Performance, Feed Utilization, Haematology, Biochemical Profile and Disease Resistance Against *Streptococcus iniae* , Aquaculture Nutrition. (2021) 27, no. 6, 1888–1902, 10.1111/anu.13326.

[11] Corradini E. , Marconcini J. M. , Agnelli J. A. , and Mattoso L. H. , Thermoplastic Blends of Corn Gluten Meal/Starch (CGM/Starch) and Corn Gluten Meal/Polyvinyl Alcohol and Corn Gluten Meal/Poly (Hydroxybutyrate-co-Hydroxyvalerate)(CGM/PHB-V), Carbohydrate Polymers. (2011) 83, no. 2, 959–965, 10.1016/j.carbpol.2010.09.004.

[12] Hernández C. , Lizárraga-Velázquez C. E. , and Contreras-Rojas D. , et al.Fish Meal Replacement by Corn Gluten in Feeds for Juvenile Spotted Rose Snapper (*Lutjanus guttatus*): Effect on Growth Performance, Feed Efficiency, Hematological Parameters, Protease Activity, Body Composition, and Nutrient Digestibility, Aquaculture. (2021) 531, 10.1016/j.aquaculture.2020.735896, 735896.

[13] Sim Y. J. , Cho S. H. , and Kwon T. W. , et al.Replacement Effects of Fish Meal by Plant Protein Sources in Diets With or Without Jack Mackerel Meal Inclusion on Growth Performance of Red Sea Bream (*Pagrus major*), Aquaculture Nutrition. (2026) 2026, no. 1, 10.1155/anu/2260317, 2260317. 41487310 PMC12759266

[14] Bai N. , Gu M. , Liu M. , Jia Q. , Pan S. , and Zhang Z. , Corn Gluten Meal Induces Enteritis and Decreases Intestinal Immunity and Antioxidant Capacity in Turbot (*Scophthalmus maximus*) at High Supplementation Levels, PLoS ONE. (2019) 14, no. 3, 10.1371/journal.pone.0213867. PMC6415862 30865702

[15] Macusi E. D. , Cayacay M. A. , and Borazon E. Q. , et al.Protein Fishmeal Replacement in Aquaculture: A Systematic Review and Implications on Growth and Adoption Viability, Sustainability. (2023) 15, no. 16, 10.3390/su151612500, 12500.

[16] Villanueva-Gutiérrez E. , González-Félix M. L. , Gatlin D. M.III, and Perez-Velazquez M. , Use of Alternative Plant and Animal Protein Blends, in Place of Fishmeal, in Diets for Juvenile Totoaba, *Totoaba macdonaldi* , Aquaculture. (2020) 529, 10.1016/j.aquaculture.2020.735698, 735698.

[17] Gyan W. R. , Yohana M. A. , Yang Q. , Tan B. , Chi S. , and Yi Y. , The Effects of Dietary L-Carnitine Supplementation in Fish Diet Containing High Corn Gluten Meal on Immunity, Lipid Metabolism, and Metabolomics in Juvenile Hybrid Grouper (♀ *Epinephelus fuscoguttatus* × ♂ *Epinephelus lanceolatus*), Aquaculture International. (2024) 32, no. 1, 833–869, 10.1007/s10499-023-01193-6.

[18] Hu L. , Yun B. , and Xue M. , et al.Effects of Fish Meal Quality and Fish Meal Substitution by Animal Protein Blend on Growth Performance, Flesh Quality and Liver Histology of Japanese Seabass (*Lateolabrax japonicus*), Aquaculture. (2013) 372–375, 52–61, 10.1016/j.aquaculture.2012.10.025.

[19] Liang X. F. , Hu L. , and Dong Y. C. , et al.Substitution of Fish Meal by Fermented Soybean Meal Affects the Growth Performance and Flesh Quality of Japanese Seabass (*Lateolabrax japonicus*), Animal Feed Science and Technology. (2017) 229, 1–12, 10.1016/j.anifeedsci.2017.03.006.

[20] Pratoomyot J. , Bendiksen E.Å. , Bell J. G. , and Tocher D. R. , Effects of Increasing Replacement of Dietary Fishmeal With Plant Protein Sources on Growth Performance and Body Lipid Composition of Atlantic Salmon (*Salmo salar* L.), Aquaculture. (2010) 305, no. 1–4, 124–132, 10.1016/j.aquaculture.2010.04.019.

[21] Ding L. , Chen J. , and Zhang Y. , et al.Effects of Dietary Fish Meal Replacement With Composite Mixture of Chicken Meal, Krill Meal, and Plant Proteins on Growth, Physiological Metabolism, and Intestinal Microbiota of Chinese Perch (*Siniperca chuatsi*), Aquaculture Nutrition. (2023) 2023, 13, 10.1155/2023/2915916, 2915916. 39553243 PMC11221970

[22] Li S. , Dai M. , Qiu H. , and Chen N. , Effects of Fishmeal Replacement With Composite Mixture of Shrimp Hydrolysate and Plant Proteins on Growth Performance, Feed Utilization, and Target of Rapamycin Pathway in Largemouth Bass, *Micropterus salmoides* , Aquaculture. (2021) 533, 10.1016/j.aquaculture.2020.736185, 736185.

[23] Cabral E. M. , Bacelar M. , Batista S. , Castro-Cunha M. , Ozório R. O. A. , and Valente L. M. P. , Replacement of Fishmeal by Increasing Levels of Plant Protein Blends in Diets for Senegalese Sole (*Solea senegalensis*) Juveniles, Aquaculture. (2011) 322–323, 74–81, 10.1016/j.aquaculture.2011.09.023.

[24] Kikuchi K. , Use of Defatted Soybean Meal as a Substitute for Fish Meal in Diets of Japanese Flounder (*Paralichthys olivaceus*), Aquaculture. (1999) 179, no. 1–4, 3–11, 10.1016/S0044-8486(99)00147-7.

[25] Karapanagiotidis I. T. , Psofakis P. , Mente E. , Malandrakis E. , and Golomazou E. , Effect of Fishmeal Replacement by Poultry By-Product Meal on Growth Performance, Proximate Composition, Digestive Enzyme Activity, Haematological Parameters and Gene Expression of Gilthead Seabream (*Sparus aurata*), Aquaculture Nutrition. (2019) 25, no. 1, 3–14, 10.1111/anu.12824.

[26] Hossain M. S. , Small B. C. , Kumar V. , and Hardy R. , Utilization of Functional Feed Additives to Produce Cost-Effective, Ecofriendly Aquafeeds High in Plant-Based Ingredients, Reviews in Aquaculture. (2024) 16, no. 1, 121–153, 10.1111/raq.12824.

[27] Ismail T. , Hegazi E. , and Dawood M. A. O. , et al.Using of Betaine to Replace Fish Meal With Soybean or/and Corn Gluten Meal in Nile Tilapia (*Oreochromis niloticus*) Diets: Histomorphology, Growth, Fatty Acid, and Glucose-Related Gene Expression Traits, Aquaculture Reports. (2020) 17, 10.1016/j.aqrep.2020.100376, 100376.

[28] Jeong H. S. , Choi D. G. , and Lee K. W. , et al.Attractiveness of Various Crude Feed Ingredients to Juvenile Olive Flounder (*Paralichthys olivaceus*, Temminck & Schlegel) and Its Application to Aquaculture, Aquaculture Research. (2020) 51, no. 11, 4517–4532, 10.1111/are.14797.

[29] Kader M. A. , Bulbul M. , and Koshio S. , et al.Effect of Complete Replacement of Fishmeal by Dehulled Soybean Meal With Crude Attractants Supplementation in Diets for Red Sea Bream, *Pagrus major* , Aquaculture. (2012) 350–353, 109–116, 10.1016/j.aquaculture.2012.04.009.

[30] Baek S. I. and Cho S. H. , Effects of Dietary Inclusion of a Crude Protein Source Exhibiting the Strongest Attractiveness to Red Sea Bream (*Pagrus major*) on Growth, Feed Availability, and Economic Efficiency, Animals. (2024) 14, no. 5, 10.3390/ani14050771, 771. 38473156 PMC10931256

[31] Takakuwa F. , Masumoto T. , and Fukada H. , Identification of Feeding Stimulants for Greater Amberjack *Seriola dumerili* in Muscle Tissue of Jack Mackerel *Trachurus japonicus* , Fisheries Science. (2019) 85, no. 2, 387–395, 10.1007/s12562-018-01285-w.

[32] Ikeda I. , Okamoto Y. , and Oda K. , Identification of Feeding Stimulants for Japanese Flounder in Muscle Extract of Jack Mackerel, Aquaculture Science. (2012) 60, 195–198, 10.11233/aquaculturesci.60.195.

[33] Hidaka I. , Kohbara J. , and Araki T. , et al.Identification of Feeding Stimulants From a Jack Mackerel Muscle Extract for Young Yellowtail *Seriola quinqueradiata* , Aquaculture. (2000) 181, no. 1-2, 115–126, 10.1016/S0044-8486(99)00221-5.

[34] KOSIS , Korean Statistical Information Service, 2025, (accessed November 15, 2025) https://kosis.kr/statHtml/statHtml.do?orgId=101&tblId=DT_1EZ0008&conn_path=I2.

[35] Biswas A. , Takahashi Y. , Isaka K. , Takakuwa F. , Tanaka H. , and Takii K. , Total Replacement of Fish Meal by the Combination of Fish Residue Meal and Soy Protein From Soymilk in the Diet of Red Sea Bream (*Pagrus major*), Animals. (2022) 12, no. 23, 10.3390/ani12233351, 3351. 36496871 PMC9740839

[36] Dossou S. , Koshio S. , and Ishikawa M. , et al.Effect of Partial Replacement of Fish Meal by Fermented Rapeseed Meal on Growth, Immune Response and Oxidative Condition of Red Sea Bream Juvenile, *Pagrus major* , Aquaculture. (2018) 490, 228–235, 10.1016/j.aquaculture.2018.02.010.

[37] Dossou S. , Koshio S. , and Ishikawa M. , et al.Effects of Replacing Fishmeal With Fermented and Non-Fermented Rapeseed Meal on the Growth, Immune and Antioxidant Responses of Red Sea Bream (*Pagrus major*), Aquaculture Nutrition. (2019) 25, no. 2, 508–517, 10.1111/anu.12876.

[38] Garling D. L.Jr. and Wilson R. P. , Optimum Dietary Protein to Energy Ratio for Channel Catfish Fingerlings, *Ictalurus punctatus* , The Journal of Nutrition. (1976) 106, no. 9, 1368–1375, 10.1093/jn/106.9.1368. 956918

[39] Takeuchi T. , Shiina Y. , and Watanabe T. , Suitable Protein and Lipid Levels in Diet for Fingerlings of Red Sea Bream *Pagrus major* , Nippon Suisan Gakkaishi. (1991) 57, no. 2, 293–299, 10.2331/suisan.57.293.

[40] Lange S. , Guđmundsdottir B. K. , and Magnadottir B. , Humoral Immune Parameters of Cultured Atlantic Halibut (*Hippoglossus hippoglossus* L.), Fish & Shellfish Immunology. (2001) 11, no. 6, 523–535, 10.1006/fsim.2000.0333. 11556481

[41] Martínez-Llorens S. , Moñino A. V. , Tomás Vidal A. , Salvador V. J. M. , Pla Torres M. , and Jover Cerdá M. , Soybean Meal as a Protein Source in Gilthead Sea Bream (*Sparus aurata* L.) Diets: Effects on Growth and Nutrient Utilization, Aquaculture Research. (2007) 38, no. 1, 82–90, 10.1111/j.1365-2109.2006.01637.x.

[42] Duncan D. B. , Multiple Range and Multiple F Tests, Biometrics. (1955) 11, no. 1, 1–42, 10.2307/3001478.

[43] Kader M. A. , Koshio S. , and Ishikawa M. , et al.Growth, Nutrient Utilization, Oxidative Condition, and Element Composition of Juvenile Red Sea Bream *Pagrus major* Fed With Fermented Soybean Meal and Scallop By-Product Blend as Fishmeal Replacement, Fisheries Science. (2011) 77, no. 1, 119–128, 10.1007/s12562-010-0312-9.

[44] Lee T. H. , Oh H. Y. , Lee G. J. , Kim H. S. , and Choi J. , Effects of Replacing Fish Meal With a Blend of Corn Gluten Meal and Meat Meal on the Growth, Biochemical Profile, Digestive Enzyme Activity, Antioxidant Status, and Innate Immunity of Juvenile Black Rockfish (*Sebastes schlegelii*), Aquaculture. (2024) 579, 10.1016/j.aquaculture.2023.740225, 740225.

[45] Kader M. A. , Koshio S. , Ishikawa M. , Yokoyama S. , and Bulbul M. , Supplemental Effects of Some Crude Ingredients in Improving Nutritive Values of Low Fishmeal Diets for Red Sea Bream, *Pagrus major* , Aquaculture. (2010) 308, no. 3-4, 136–144, 10.1016/j.aquaculture.2010.07.037.

[46] Wei Y. , Shen H. , and Xu W. , et al.Replacement of Dietary Fishmeal by Antarctic Krill Meal on Growth Performance, Intestinal Morphology, Body Composition and Organoleptic Quality of Large Yellow Croaker *Larimichthys crocea* , Aquaculture. (2019) 512, 10.1016/j.aquaculture.2019.734281, 734281.

[47] Gaber M. M. A. , The Effect of Different Levels of Krill Meal Supplementation of Soybean-Based Diets on Feed Intake, Digestibility, and Chemical Composition of Juvenile Nile Tilapia *Oreochromis niloticus*, L, Journal of the World Aquaculture Society. (2005) 36, no. 3, 346–353, 10.1111/j.1749-7345.2005.tb00338.x.

[48] Gunathilaka B. E. , Jeong S. , and Cho M. , et al.Effects of Dietary Fish Meal Replacement With Alternative Protein Ingredients and Their Combinations on Growth Performance, Feed Utilization, Fillet Composition, and Biochemical Parameters of Red Seabream (*Pagrus major*), Aquaculture Nutrition. (2023) 2023, 16, 10.1155/2023/8883739, 8883739. 37483331 PMC10359139

[49] Koshio S. , Webster C. D. and Lim C. , Red Sea Bream, *Pagrus major* , Nutrient Requirements and Feeding of Finfish for Aquaculture, 2002, CABI, Wallingford, United Kingdom, 51–63.

[50] Forster I. and Ogata H. Y. , Lysine Requirement of Juvenile Japanese Flounder *Paralichthys olivaceus* and Juvenile Red Sea Bream *Pagrus major* , Aquaculture. (1998) 161, no. 1–4, 131–142, 10.1016/S0044-8486(97)00263-9.

[51] Rahimnejad S. and Lee K.-J. , Dietary Valine Requirement of Juvenile Red Sea Bream *Pagrus major* , Aquaculture. (2013) 416–417, 212–218, 10.1016/j.aquaculture.2013.09.026.

[52] Rahimnejad S. and Lee K.-J. , Dietary Arginine Requirement of Juvenile Red Sea Bream *Pagrus major* , Aquaculture. (2014) 434, 418–424, 10.1016/j.aquaculture.2014.09.003.

[53] Zhou Z. , Wang X. , and Wu X. , et al.Effects of Dietary Leucine Levels on Growth, Feed Utilization, Neuro-Endocrine Growth Axis and TOR-Related Signaling Genes Expression of Juvenile Hybrid Grouper (*Epinephelus fuscoguttatus* ♀ × *Epinephelus lanceolatus* ♂), Aquaculture. (2019) 504, 172–181, 10.1016/j.aquaculture.2019.02.005.

[54] Zehra S. and Yousif R. A. , Dietary Total Aromatic Amino Acid Requirement and Tyrosine Replacement Value for Phenylalanine for Fingerling *Oreochromis niloticus* (Linnaeus), Aquaculture Nutrition. (2021) 27, no. 4, 1009–1018, 10.1111/anu.13242.

[55] Gan L. , Zhou L.-L. , Li X.-X. , and Yue Y.-R. , Dietary Leucine Requirement of Juvenile Nile Tilapia, *Oreochromis niloticus* , Aquaculture Nutrition. (2016) 22, no. 5, 1040–1046, 10.1111/anu.12353.

[56] Xiao W. , Zou Z. , Li D. , Zhu J. , Yue Y. , and Yang H. , Effect of Dietary Phenylalanine Level on Growth Performance, Body Composition, and Biochemical Parameters in Plasma of Juvenile Hybrid Tilapia, *Oreochromis niloticus* × *Oreochromis aureus* , Journal of the World Aquaculture Society. (2020) 51, no. 2, 437–451, 10.1111/jwas.12641.

[57] Jin M. , Lu Y. , and Yuan Y. , et al.Regulation of Growth, Antioxidant Capacity, Fatty Acid Profiles, Hematological Characteristics and Expression of Lipid Related Genes by Different Dietary n–3 Highly Unsaturated Fatty Acids in Juvenile Black Seabream (*Acanthopagrus schlegelii*), Aquaculture. (2017) 471, 55–65, 10.1016/j.aquaculture.2017.01.004. PMC5400258 28430821

[58] Li M. , Xu C. , and Ma Y. , et al.Effects of Dietary n–3 Highly Unsaturated Fatty Acids Levels on Growth, Lipid Metabolism and Innate Immunity in Juvenile Golden Pompano (*Trachinotus ovatus*), Fish & Shellfish Immunology. (2020) 105, 177–185, 10.1016/j.fsi.2020.06.060. 32634552

[59] Yeşilayer N. and Kaymak I. E. , Effect of Partial Replacement of Dietary Fish Meal by Soybean Meal With Betaine Attractant Supplementation on Growth Performance and Fatty Acid Profiles of Juvenile Rainbow Trout (*Oncorhynchus mykiss*), Aquaculture Research. (2020) 51, no. 4, 1533–1541, 10.1111/are.14501.

[60] Bharti S. and Rasool F. , Analysis of the Biochemical and Histopathological Impact of a Mild Dose of Commercial Malathion on *Channa punctatus* (Bloch) Fish, Toxicology Reports. (2021) 8, 443–455, 10.1016/j.toxrep.2021.02.018. 33717997 PMC7933801

[61] Timmerhaus G. , Lazado C. C. , Cabillon N. A. R. , Reiten B. K. M. , and Johansen L. , The Optimum Velocity for Atlantic Salmon Post-Smolts in RAS Is a Compromise Between Muscle Growth and Fish Welfare, Aquaculture. (2021) 532, 10.1016/j.aquaculture.2020.736076, 736076.

[62] Sivaramakrishnan T. , Ambasankar K. , and Kumar T. S. , et al.Influence of Dietary Protein Levels on Growth, Feed Utilization, Body Indices and Serum Profile of Silver Moony *Monodactylus argenteus* , Aquaculture. (2022) 549, 10.1016/j.aquaculture.2021.737823, 737823.

[63] Dernekbaşı S. , Digestibility and Liver Fatty Acid Composition of Rainbow Trout (*Oncorhynchus mykiss*) Fed by Graded Levels of Canola Oil, Turkish Journal of Fisheries and Aquatic Sciences. (2012) 12, no. 1, 105–113, 10.4194/1303-2712-v12_1_13.

[64] Monge-Ortiz R. , Tomás-Vidal A. , and Gallardo-Álvarez F. J. , et al.Partial and Total Replacement of Fishmeal by a Blend of Animal and Plant Proteins in Diets for *Seriola dumerili*: Effects on Performance and Nutrient Efficiency, Aquaculture Nutrition. (2018) 24, no. 4, 1163–1174, 10.1111/anu.12655.

[65] Sankian Z. , Khosravi S. , Kim Y.-O. , and Lee S.-M. , Effects of Dietary Inclusion of Yellow Mealworm (*Tenebrio molitor*) Meal on Growth Performance, Feed Utilization, Body Composition, Plasma Biochemical Indices, Selected Immune Parameters and Antioxidant Enzyme Activities of Mandarin Fish (*Siniperca scherzeri*) Juveniles, Aquaculture. (2018) 496, 79–87, 10.1016/j.aquaculture.2018.07.012.

[66] Luo C. , Gwekwe B. , and Choto P. , et al.Bitter Peptides From Enzymatically Hydrolyzed Protein Increase the Number of Leucocytes and Lysozyme Activity of Large Yellow Croaker (*Larimichthys crocea*), Fish & Shellfish Immunology. (2018) 81, 130–134, 10.1016/j.fsi.2018.07.013. 30006041

[67] Liu T. , Han T. , and Wang J. , et al.Effects of Replacing Fish Meal With Soybean Meal on Growth Performance, Feed Utilization and Physiological Status of Juvenile Redlip Mullet *Liza haematocheila* , Aquaculture Reports. (2021) 20, 10.1016/j.aqrep.2021.100756, 100756.

[68] Jeong H. S. , Kim J. , Olowe O. S. , and Cho S. H. , Dietary Optimum Inclusion Level of Jack Mackerel Meal for Olive Flounder (*Paralichthys olivaceus*, Temminck & Schlegel, 1846), Aquaculture. (2022) 559, 10.1016/j.aquaculture.2022.738432, 738432.

[69] Cai W. , Jiang G. , and Li X. , et al.Effects of Complete Fish Meal Replacement by Rice Protein Concentrate With or Without Lysine Supplement on Growth Performance, Muscle Development and Flesh Quality of Blunt Snout Bream (*Megalobrama amblycephala*), Aquaculture Nutrition. (2018) 24, no. 1, 481–491, 10.1111/anu.12581.

[70] Kaushik S. J. and Seiliez I. , Protein and Amino Acid Nutrition and Metabolism in Fish: Current Knowledge and Future Needs, Aquaculture Research. (2010) 41, no. 3, 322–332, 10.1111/j.1365-2109.2009.02174.x.

[71] Sobczak M. , Panicz R. , and Eljasik P. , et al.Quality Improvement of Common Carp (*Cyprinus carpio* L.) Meat Fortified With n–3 PUFA, Food and Chemical Toxicology. (2020) 139, 10.1016/j.fct.2020.111261, 111261. 32198031

[72] Wang X. , Dong Y. , and Huang Y. , et al.Docosahexaenoic Acid-Enriched Diet Improves the Flesh Quality of Freshwater Fish (*Megalobrama amblycephala*): Evaluation Based on Nutritional Value, Texture and Flavor, Food Chemistry. (2024) 460, 10.1016/j.foodchem.2024.140518, 140518. 39047487

[73] Gasco L. , Henry M. , and Piccolo G. , et al. *Tenebrio molitor* Meal in Diets for European Sea Bass (*Dicentrarchus labrax* L.) Juveniles: Growth Performance, Whole Body Composition and In Vivo Apparent Digestibility, Animal Feed Science and Technology. (2016) 220, 34–45, 10.1016/j.anifeedsci.2016.07.003.

[74] Cabral E. M. , Fernandes T. J. R. , and Campos S. D. , et al.Replacement of Fish Meal by Plant Protein Sources up to 75% Induces Good Growth Performance Without Affecting Flesh Quality in Ongrowing Senegalese Sole, Aquaculture. (2013) 380, 130–138, 10.1016/j.aquaculture.2012.12.006.

