# Supplementary material for: Inclusion of Jack Mackerel Meal in Low‐Fish Meal Diets: Impacts on the Growth and Feed Utilization of Red Sea Bream (Pagrus major) and Economic Analysis
Source: Aquac Nutr. 2026 Jul 2;2026:5589868. doi: 10.1155/anu/5589868 (PMC13325012; doi:10.1155/anu/5589868)
Supplement: Supplementary file 1 — Supporting Information Table S1: Amino acid (% of the diet) profiles of the main protein ingredients and the experimental diets. Table S2: Fatty acid (% of total fatty acids) profiles of the main protein ingredients and the experimental diets. Table S3: Amino acid (% of wet weight) profiles of the whole‐body red sea bream fed the experimental diets for 8 weeks. Table S4: Fatty acid (% of total fatty acids) profiles of the whole‐body red sea bream fed the experimental diets for 8 weeks. [file ANU-2026-5589868-s001.docx]

**Table S1**

|  | Main protein ingredients | | |  |  |  | Experimental diets | | | | |
| --- | --- | --- | --- | --- | --- | --- | --- | --- | --- | --- | --- |
|  | FM | JMM | MC |  | Requirement |  | Con | MC25 | MC50 | MC25J | MC50J |
| *Indispensable amino acids (IAA) (%)* | | | | | | | | | | | |
| Arginine | 4.24 | 4.02 | 3.90 |  | 2.37^1^ |  | 2.88 | 2.82 | 2.76 | 2.73 | 2.69 |
| Histidine | 2.00 | 2.07 | 1.43 |  |  |  | 1.34 | 1.29 | 1.20 | 1.32 | 1.25 |
| Isoleucine | 3.02 | 2.70 | 2.25 |  |  |  | 2.09 | 1.90 | 1.82 | 1.79 | 1.73 |
| Leucine | 5.47 | 5.01 | 7.64 |  |  |  | 3.76 | 4.39 | 4.78 | 4.27 | 4.56 |
| Lysine | 5.80 | 5.48 | 2.67 |  | 1.79^2^ |  | 3.84 | 3.56 | 3.03 | 3.50 | 2.99 |
| Phenylalanine | 2.93 | 2.62 | 3.27 |  |  |  | 2.17 | 2.18 | 2.20 | 2.10 | 2.18 |
| Threonine | 3.14 | 2.98 | 2.31 |  |  |  | 2.19 | 2.05 | 1.95 | 2.00 | 1.94 |
| Tryptophan | 0.54 | 0.53 | 0.30 |  |  |  | 0.42 | 0.40 | 0.39 | 0.39 | 0.35 |
| Valine | 3.61 | 3.22 | 3.01 |  | 0.90^3^ |  | 2.54 | 2.50 | 2.42 | 2.43 | 2.31 |
| ∑IAA^4^ | 30.75 | 28.63 | 26.78 |  |  |  | 21.23 | 21.09 | 20.55 | 20.53 | 20.00 |
| *Dispensable amino acids (DAA) (%)* | | | | | | | | | | | |
| Alanine | 4.59 | 4.42 | 6.24 |  |  |  | 2.95 | 3.31 | 3.51 | 3.30 | 3.46 |
| Aspartic acid | 6.65 | 6.21 | 4.83 |  |  |  | 4.73 | 4.63 | 4.38 | 4.57 | 4.35 |
| Glutamic acid | 9.29 | 8.70 | 11.64 |  |  |  | 7.18 | 7.69 | 8.03 | 7.56 | 7.94 |
| Glycine | 4.34 | 4.39 | 7.40 |  |  |  | 2.89 | 3.34 | 3.68 | 3.37 | 3.75 |
| Proline | 3.00 | 2.97 | 7.44 |  |  |  | 2.24 | 2.73 | 2.81 | 2.64 | 2.70 |
| Serine | 2.90 | 2.73 | 3.19 |  |  |  | 2.11 | 2.13 | 2.17 | 2.10 | 2.15 |
| Tyrosine | 2.30 | 1.83 | 2.20 |  |  |  | 1.50 | 1.27 | 1.24 | 1.25 | 1.15 |
| ∑DAA^5^ | 33.07 | 31.25 | 42.94 |  |  |  | 23.60 | 25.10 | 25.82 | 24.79 | 25.50 |

Amino acid (% of the diet) profiles of the main protein ingredients and the experimental diets.

FM: fish meal; JMM: jack mackerel meal; MC: combined meat meal and corn gluten meal; Con: the 60% FM-based diet; MC25: dietary 25% substitution of FM with MC; MC50: dietary 50% substitution of FM with MC; MC25J: MC25 with JMM inclusion; MC50J: MC50 with JMM inclusion.

^1^ Arginine, ^2^ lysine, and ^3^ valine requirements were taken from Rahimnejad & Lee (2014), Forster & Ogata (1998), and Rahimnejad & Lee (2013), respectively.

^4^ ∑IAA: total indispensable amino acids.

^5^ ∑DAA: total dispensable amino acids.

**Table S2**

|  | Main protein ingredients | | |  | Experimental diets | | | | |
| --- | --- | --- | --- | --- | --- | --- | --- | --- | --- |
|  | FM | JMM | MC |  | Con | MC25 | MC50 | MC25J | MC50J |
| C14:0 | 6.36 | 5.27 | 1.10 |  | 2.70 | 2.45 | 2.13 | 2.31 | 2.00 |
| C16:0 | 21.40 | 19.40 | 20.60 |  | 14.83 | 14.54 | 14.33 | 14.15 | 13.96 |
| C18:0 | 3.83 | 6.33 | 7.74 |  | 3.80 | 4.19 | 4.63 | 4.51 | 4.95 |
| ∑SFA^1^ | 31.59 | 31.00 | 29.44 |  | 21.33 | 21.18 | 21.09 | 20.97 | 20.91 |
| C16:1n-7 | 7.04 | 7.06 | 1.48 |  | 3.10 | 2.92 | 2.59 | 2.95 | 2.63 |
| C17:1n-7 | 1.13 | 1.05 | 0.23 |  | 0.50 | 0.42 | 0.33 | 0.39 | 0.30 |
| C18:1n-9 | 16.44 | 21.41 | 33.36 |  | 21.82 | 23.06 | 24.40 | 23.77 | 24.89 |
| C20:1n-9 | 3.49 | 1.50 | 0.65 |  | 2.69 | 2.50 | 2.49 | 2.31 | 2.24 |
| C22:1n-9 | 0.16 | 0.21 | 0.18 |  | 0.30 | 0.33 | 0.35 | 0.36 | 0.39 |
| C24:1n-9 | 3.25 | 3.64 | 0.07 |  | 1.38 | 1.02 | 0.75 | 1.11 | 0.82 |
| ∑MUFA^2^ | 31.51 | 34.87 | 35.96 |  | 29.79 | 30.25 | 30.91 | 30.89 | 31.27 |
| C18:2n-6 | 4.88 | 2.29 | 28.97 |  | 29.27 | 31.73 | 33.44 | 30.63 | 32.76 |
| C18:3n-3 | 0.36 | 0.63 | 1.22 |  | 3.49 | 4.10 | 4.18 | 4.23 | 4.32 |
| C18:3n-6 | 0.29 | 0.25 | 0.34 |  | 0.55 | 0.58 | 0.60 | 0.56 | 0.57 |
| C20:4n-6 | 2.04 | 2.51 | 0.10 |  | 2.02 | 1.88 | 1.72 | 1.99 | 1.81 |
| C20:5n-3 | 14.96 | 11.60 | 0.11 |  | 6.37 | 4.89 | 3.43 | 4.36 | 3.18 |
| C22:2n-6 | 0.53 | 0.62 | 0.23 |  | 0.32 | 0.30 | 0.26 | 0.32 | 0.28 |
| C22:6n-3 | 9.06 | 12.11 | 0.06 |  | 4.25 | 3.22 | 2.59 | 3.62 | 2.81 |
| ∑n-3 HUFA^3^ | 24.02 | 23.71 | 0.17 |  | 10.62 | 8.11 | 6.02 | 7.98 | 5.99 |
| Unknown | 4.78 | 4.12 | 3.56 |  | 2.61 | 1.87 | 1.78 | 2.43 | 2.09 |

Fatty acid (% of total fatty acids) profiles of the main protein ingredients and the experimental diets.

FM: fish meal; JMM: jack mackerel meal; MC: combined meat meal and corn gluten meal; Con: the 60% FM-based diet; MC25: dietary 25% substitution of FM with MC; MC50: dietary 50% substitution of FM with MC; MC25J: MC25 with JMM inclusion; MC50J: MC50 with JMM inclusion.

^1^ ∑SFA: total saturated fatty acids.

^2^ ∑MUFA: total monounsaturated fatty acids.

^3^ ∑n-3 HUFA: total n-3 highly unsaturated fatty acids.

**Table S3**

Amino acid (% of wet weight) profiles of the whole-body red sea bream fed the experimental diets for 8 weeks.

|  | Experimental diets | | | | |  |  | Main effect: FMSL | |  | Main effect: JMM inclusion | |  | Two-way ANOVA | | |  |
| --- | --- | --- | --- | --- | --- | --- | --- | --- | --- | --- | --- | --- | --- | --- | --- | --- | --- |
|  | Con | MC25 | MC50 | MC25J | MC50J | *p*-value |  | 25% | 50% |  | Without | With |  | FMSL | JMM inclusion | Interaction |  |
| *Indispensable amino acids (IAA) (%)* | | | | | | | | | | | | | | | | | |
| Arginine | 0.95 ± 0.020 | 0.94 ± 0.026 | 0.91 ± 0.020 | 0.92 ± 0.026 | 0.89 ± 0.026 | *p* > 0.4 |  | 0.93 ± 0.017 | 0.90 ± 0.015 |  | 0.92 ± 0.016 | 0.91 ± 0.018 |  | *p* > 0.2 | *p* > 0.5 | *p* > 0.8 |  |
| Histidine | 0.35 ± 0.029 | 0.35 ± 0.023 | 0.32 ± 0.035 | 0.35 ± 0.023 | 0.37 ± 0.017 | *p* > 0.7 |  | 0.35 ± 0.015 | 0.35 ± 0.021 |  | 0.34 ± 0.020 | 0.36 ± 0.014 |  | *p* > 0.8 | *p* > 0.3 | *p* > 0.3 |  |
| Isoleucine | 0.56 ± 0.029 | 0.55 ± 0.020 | 0.54 ± 0.026 | 0.52 ± 0.020 | 0.52 ± 0.023 | *p* > 0.7 |  | 0.54 ± 0.014 | 0.53 ± 0.016 |  | 0.55 ± 0.015 | 0.52 ± 0.014 |  | *p* > 0.8 | *p* > 0.3 | *p* > 0.9 |  |
| Leucine | 1.08 ± 0.023 | 1.09 ± 0.020 | 1.12 ± 0.026 | 1.08 ± 0.029 | 1.09 ± 0.026 | *p* > 0.8 |  | 1.09 ± 0.016 | 1.11 ± 0.017 |  | 1.11 ± 0.016 | 1.09 ± 0.018 |  | *p* > 0.4 | *p* > 0.4 | *p* > 0.8 |  |
| Lysine | 1.24 ± 0.026 | 1.22 ± 0.023 | 1.17 ± 0.020 | 1.19 ± 0.032 | 1.16 ± 0.032 | *p* > 0.2 |  | 1.21 ± 0.019 | 1.17 ± 0.017 |  | 1.19 ± 0.018 | 1.18 ± 0.021 |  | *p* > 0.1 | *p* > 0.5 | *p* > 0.6 |  |
| Phenylalanine | 0.59 ± 0.023 | 0.59 ± 0.026 | 0.62 ± 0.029 | 0.57 ± 0.023 | 0.59 ± 0.029 | *p* > 0.7 |  | 0.58 ± 0.016 | 0.61 ± 0.019 |  | 0.60 ± 0.019 | 0.58 ± 0.017 |  | *p* > 0.3 | *p* > 0.4 | *p* > 0.8 |  |
| Threonine | 0.68 ± 0.023 | 0.66 ± 0.017 | 0.63 ± 0.026 | 0.65 ± 0.032 | 0.62 ± 0.026 | *p* > 0.4 |  | 0.66 ± 0.016 | 0.62 ± 0.017 |  | 0.64 ± 0.016 | 0.64 ± 0.020 |  | *p* > 0.2 | *p* > 0.7 | *p* > 0.9 |  |
| Tryptophan | 0.11 ± 0.012 | 0.10 ± 0.015 | 0.08 ± 0.012 | 0.08 ± 0.015 | 0.07 ± 0.015 | *p* > 0.2 |  | 0.09 ± 0.010 | 0.07 ± 0.009 |  | 0.09 ± 0.009 | 0.08 ± 0.010 |  | *p* > 0.2 | *p* > 0.3 | *p* > 0.9 |  |
| Valine | 0.67 ± 0.029 | 0.68 ± 0.032 | 0.65 ± 0.020 | 0.67 ± 0.020 | 0.64 ± 0.026 | *p* > 0.8 |  | 0.68 ± 0.017 | 0.65 ± 0.015 |  | 0.66 ± 0.018 | 0.66 ± 0.016 |  | *p* > 0.2 | *p* > 0.8 | *p* > 0.9 |  |
| *Dispensable amino acids (DAA) (%)* | | | | | | | | | | | | | | | | | |
| Alanine | 1.09 ± 0.020 | 1.11 ± 0.017 | 1.16 ± 0.023 | 1.11 ± 0.026 | 1.13 ± 0.017 | *p* > 0.2 |  | 1.11 ± 0.014 | 1.15 ± 0.015 |  | 1.14 ± 0.017 | 1.12 ± 0.015 |  | *p* > 0.1 | *p* > 0.4 | *p* > 0.5 |  |
| Aspartic acid | 1.43 ± 0.029 | 1.43 ± 0.029 | 1.42 ± 0.026 | 1.42 ± 0.020 | 1.40 ± 0.035 | *p* > 0.9 |  | 1.42 ± 0.016 | 1.41 ± 0.020 |  | 1.43 ± 0.017 | 1.41 ± 0.018 |  | *p* > 0.6 | *p* > 0.5 | *p* > 0.8 |  |
| Glutamic acid | 2.10 ± 0.026 | 2.13 ± 0.026 | 2.15 ± 0.026 | 2.12 ± 0.029 | 2.14 ± 0.026 | *p* > 0.7 |  | 2.12 ± 0.017 | 2.15 ± 0.017 |  | 2.14 ± 0.018 | 2.13 ± 0.018 |  | *p* > 0.3 | *p* > 0.7 | *p* > 0.9 |  |
| Glycine | 1.24 ± 0.032 | 1.25 ± 0.026 | 1.27 ± 0.026 | 1.26 ± 0.032 | 1.29 ± 0.029 | *p* > 0.8 |  | 1.26 ± 0.018 | 1.28 ± 0.018 |  | 1.26 ± 0.017 | 1.27 ± 0.021 |  | *p* > 0.4 | *p* > 0.6 | *p* > 0.7 |  |
| Proline | 0.74 ± 0.026 | 0.80 ± 0.023 | 0.83 ± 0.015 | 0.78 ± 0.020 | 0.82 ± 0.020 | *p* > 0.1 |  | 0.79 ± 0.014 | 0.82 ± 0.011 |  | 0.81 ± 0.014 | 0.80 ± 0.015 |  | *p* > 0.1 | *p* > 0.5 | *p* > 0.8 |  |
| Serine | 0.69 ± 0.026 | 0.70 ± 0.020 | 0.72 ± 0.020 | 0.69 ± 0.023 | 0.67 ± 0.020 | *p* > 0.7 |  | 0.70 ± 0.014 | 0.70 ± 0.016 |  | 0.71 ± 0.013 | 0.68 ± 0.014 |  | *p* > 0.9 | *p* > 0.2 | *p* > 0.4 |  |
| Tyrosine | 0.43 ± 0.020 | 0.43 ± 0.026 | 0.41 ± 0.020 | 0.41 ± 0.023 | 0.38 ± 0.023 | *p* > 0.5 |  | 0.42 ± 0.016 | 0.40 ± 0.016 |  | 0.42 ± 0.015 | 0.40 ± 0.016 |  | *p* > 0.3 | *p* > 0.3 | *p* > 0.7 |  |

Con: the 60% fish meal (FM)-based diet; MC25: dietary 25% substitution of FM with combined meat meal and corn gluten meal (MC); MC50: dietary 50% substitution of FM with MC; MC25J: MC25 with jack mackerel meal (JMM) inclusion; MC50J: MC50 with JMM inclusion; FMSL: FM substitution level.

Values are expressed as means of triplicates ± SE.

**Table S4**

Fatty acid (% of total fatty acids) profiles of the whole-body red sea bream fed the experimental diets for 8 weeks.

|  | Experimental diets | | | | |  |  | Main effect: FMSL | |  | Main effect: JMM inclusion | |  | Two-way ANOVA | | |
| --- | --- | --- | --- | --- | --- | --- | --- | --- | --- | --- | --- | --- | --- | --- | --- | --- |
|  | Con | MC25 | MC50 | MC25J | MC50J | *p*-value |  | 25% | 50% |  | Without | With |  | FMSL | JMM inclusion | Interaction |
| C14:0 | 2.12 ± 0.049^a^ | 1.89 ± 0.032^b^ | 1.84 ± 0.020^bc^ | 1.80 ± 0.029^bc^ | 1.77 ± 0.026^c^ | *p* < 0.0001 |  | 1.84 ± 0.027 | 1.81 ± 0.020 |  | 1.86 ± 0.020^A^ | 1.79 ± 0.018^B^ |  | *p* > 0.1 | *p* < 0.03 | *p* > 0.6 |
| C16:0 | 14.24 ± 0.066^a^ | 14.07 ± 0.043^b^ | 13.93 ± 0.055^bc^ | 13.80 ± 0.078^bc^ | 13.68 ± 0.141^c^ | *p* < 0.007 |  | 13.94 ± 0.072 | 13.81 ± 0.088 |  | 14.00 ± 0.044^A^ | 13.74 ± 0.077^B^ |  | *p* > 0.1 | *p* < 0.02 | *p* > 0.9 |
| C18:0 | 5.19 ± 0.069^d^ | 5.57 ± 0.038^c^ | 5.71 ± 0.032^b^ | 5.80 ± 0.024^b^ | 6.00 ± 0.049^a^ | *p* < 0.0001 |  | 5.68 ± 0.055^B^ | 5.86 ± 0.069^A^ |  | 5.64 ± 0.039^B^ | 5.90 ± 0.051^A^ |  | *p* < 0.002 | *p* < 0.0001 | *p* > 0.4 |
| ∑SFA^1^ | 21.54 ± 0.052 | 21.53 ± 0.103 | 21.48 ± 0.043 | 21.40 ± 0.084 | 21.45 ± 0.118 | *p* > 0.7 |  | 21.47 ± 0.066 | 21.47 ± 0.057 |  | 21.51 ± 0.057 | 21.43 ± 0.066 |  | *p* > 0.9 | *p* > 0.4 | *p* > 0.5 |
| C16:1n-7 | 3.38 ± 0.090^a^ | 3.16 ± 0.026^b^ | 3.04 ± 0.038^b^ | 3.13 ± 0.020^b^ | 3.03 ± 0.038^b^ | *p* < 0.003 |  | 3.15 ± 0.016^A^ | 3.04 ± 0.024^B^ |  | 3.10 ± 0.034 | 3.08 ± 0.029 |  | *p* < 0.008 | *p* > 0.6 | *p* > 0.7 |
| C17:1n-7 | 0.41 ± 0.026 | 0.38 ± 0.020 | 0.36 ± 0.023 | 0.36 ± 0.026 | 0.35 ± 0.026 | *p* > 0.4 |  | 0.37 ± 0.015 | 0.35 ± 0.016 |  | 0.37 ± 0.014 | 0.36 ± 0.017 |  | *p* > 0.5 | *p* > 0.5 | *p* > 0.9 |
| C18:1n-9 | 24.91 ± 0.309^d^ | 25.68 ± 0.147^c^ | 26.01 ± 0.098^c^ | 27.01 ± 0.078^b^ | 27.58 ± 0.098^a^ | *p* < 0.0001 |  | 26.35 ± 0.307^B^ | 26.80 ± 0.357^A^ |  | 25.85 ± 0.108^B^ | 27.30 ± 0.139^A^ |  | *p* < 0.003 | *p* < 0.0001 | *p* > 0.3 |
| C20:1n-9 | 2.74 ± 0.061^a^ | 2.64 ± 0.035^ab^ | 2.58 ± 0.040^bc^ | 2.47 ± 0.020^cd^ | 2.42 ± 0.040^d^ | *p* < 0.002 |  | 2.56 ± 0.041 | 2.50 ± 0.044 |  | 2.61 ± 0.027^A^ | 2.45 ± 0.023^B^ |  | *p* > 0.1 | *p* < 0.002 | *p* > 0.9 |
| C22:1n-9 | 0.36 ± 0.026 | 0.36 ± 0.012 | 0.37 ± 0.018 | 0.38 ± 0.018 | 0.39 ± 0.017 | *p* > 0.7 |  | 0.37 ± 0.011 | 0.38 ± 0.012 |  | 0.37 ± 0.10 | 0.39 ± 0.011 |  | *p* > 0.5 | *p* > 0.2 | *p* > 0.8 |
| C24:1n-9 | 1.69 ± 0.038^a^ | 1.40 ± 0.026^bc^ | 1.31 ± 0.038^c^ | 1.43 ± 0.032^b^ | 1.35 ± 0.032^bc^ | *p* < 0.0001 |  | 1.42 ± 0.019^A^ | 1.33 ± 0.024^B^ |  | 1.36 ± 0.029 | 1.39 ± 0.026 |  | *p* < 0.04 | *p* > 0.3 | *p* > 0.8 |
| ∑MUFA^2^ | 33.49 ± 0.121^b^ | 33.62 ± 0.208^b^ | 33.67 ± 0.049^b^ | 34.79 ± 0.078^a^ | 35.12 ± 0.163^a^ | *p* < 0.0001 |  | 34.21 ± 0.281 | 34.40 ± 0.333 |  | 33.65 ± 0.096^B^ | 34.96 ± 0.109^A^ |  | *p* > 0.2 | *p* < 0.0001 | *p* > 0.3 |
| C18:2n-6 | 28.17 ± 0.133^c^ | 30.03 ± 0.147^a^ | 30.21 ± 0.121^a^ | 29.09 ± 0.170^b^ | 29.31 ± 0.081^b^ | *p* < 0.0001 |  | 29.56 ± 0.232 | 29.76 ± 0.212 |  | 30.12 ± 0.095^A^ | 29.20 ± 0.097^B^ |  | *p* > 0.1 | *p* < 0.0001 | *p* > 0.9 |
| C18:3n-3 | 2.96 ± 0.043^c^ | 2.97 ± 0.032^c^ | 3.00 ± 0.020^bc^ | 3.07 ± 0.020^ab^ | 3.16 ± 0.029^a^ | *p* < 0.004 |  | 3.02 ± 0.028 | 3.08 ± 0.038 |  | 2.99 ± 0.018^B^ | 3.12 ± 0.025^A^ |  | *p* > 0.05 | *p* < 0.001 | *p* > 0.3 |
| C18:3n-6 | 0.56 ± 0.038 | 0.64 ± 0.020 | 0.65 ± 0.026 | 0.60 ± 0.017 | 0.63 ± 0.029 | *p* > 0.2 |  | 0.62 ± 0.015 | 0.64 ± 0.018 |  | 0.65 ± 0.015 | 0.62 ± 0.016 |  | *p* > 0.5 | *p* > 0.2 | *p* > 0.5 |
| C20:4n-6 | 1.74 ± 0.090 | 1.67 ± 0.035 | 1.62 ± 0.020 | 1.70 ± 0.022 | 1.65 ± 0.040 | *p* > 0.4 |  | 1.69 ± 0.019 | 1.63 ± 0.022 |  | 1.64 ± 0.022 | 1.68 ± 0.023 |  | *p* > 0.1 | *p* > 0.3 | *p* > 0.9 |
| C20:5n-3 | 4.36 ± 0.029^a^ | 3.11 ± 0.035^b^ | 3.00 ± 0.032^c^ | 2.95 ± 0.027^c^ | 2.85 ± 0.035^d^ | *p* < 0.0001 |  | 3.03 ± 0.042^A^ | 2.92 ± 0.040^B^ |  | 3.05 ± 0.033^A^ | 2.90 ± 0.030^B^ |  | *p* < 0.01 | *p* < 0.001 | *p* > 0.8 |
| C22:2n-6 | 0.41 ± 0.038 | 0.35 ± 0.020 | 0.30 ± 0.032 | 0.37 ± 0.017 | 0.32 ± 0.015 | *p* > 0.09 |  | 0.36 ± 0.013 | 0.31 ± 0.017 |  | 0.32 ± 0.020 | 0.35 ± 0.015 |  | *p* > 0.05 | *p* > 0.2 | *p* > 0.9 |
| C22:6n-3 | 4.03 ± 0.061^a^ | 3.68 ± 0.040^bc^ | 3.54 ± 0.026^c^ | 3.82 ± 0.063^b^ | 3.65 ± 0.033^c^ | *p* < 0.0001 |  | 3.75 ± 0.046^A^ | 3.59 ± 0.031^B^ |  | 3.61 ± 0.039^B^ | 3.74 ± 0.051^A^ |  | *p* < 0.006 | *p* < 0.002 | *p* > 0.7 |
| ∑n-3 HUFA^3^ | 8.39 ± 0.032^a^ | 6.79 ± 0.075^b^ | 6.53 ± 0.058^c^ | 6.77 ± 0.036^b^ | 6.49 ± 0.046^c^ | *p* < 0.0001 |  | 6.78 ± 0.038^A^ | 6.51 ± 0.034^B^ |  | 6.66 ± 0.071 | 6.63 ± 0.067 |  | *p* < 0.001 | *p* > 0.6 | *p* > 0.8 |
| Unknown | 2.75 ± 0.188 | 2.40 ± 0.301 | 2.54 ± 0.125 | 2.20 ± 0.340 | 1.86 ± 0.214 |  |  |  |  |  |  |  |  |  |  |  |

Con: the 60% fish meal (FM)-based diet; MC25: dietary 25% substitution of FM with combined meat meal and corn gluten meal (MC); MC50: dietary 50% substitution of FM with MC; MC25J: MC25 with jack mackerel meal (JMM) inclusion; MC50J: MC50 with JMM inclusion; FMSL: FM substitution level.

Values are expressed as means of triplicates ± SE. Significant differences (*p* < 0.05) were identified using Duncan’s multiple range test (lowercase letters) and two-way ANOVA (uppercase letters).

^1^ ∑SFA: total saturated fatty acids.

^2^ ∑MUFA: total monounsaturated fatty acids.

^3^ ∑n-3 HUFA: total n-3 highly unsaturated fatty acids.
